# Advancing total synthesis through the Stille cross-coupling: recent innovations and case studies

**DOI:** 10.1039/d5ra06557g

**Published:** 2026-01-12

**Authors:** Elaheh Hashemi, Mohammad Teimoury

**Affiliations:** a Department of Chemistry, Faculty of Sciences, Shahid Rajaee Teacher Training University P.O. Box: 1678815811 Tehran Iran e.hashemi@sru.ac.ir

## Abstract

The Stille reaction, a pivotal palladium-catalyzed cross-coupling, continues to drive advancements in the total synthesis of highly complex natural products, pharmaceuticals, and functional materials. Originating from *John K. Stille*'s foundational work, this method has evolved through innovations in catalysis, ligand design, and eco-friendly protocols to address challenges like tin toxicity. This review highlights its application in constructing complex molecules and emphasizes advancements in bond-forming strategies. The reaction's versatility in both intermolecular and intramolecular contexts, coupled with its functional group tolerance, underscores its enduring relevance in modern organic synthesis of naural products.

## Abstract 2

The Stille reaction, a pivotal palladium-catalyzed cross-coupling, continues to drive advancements in the total synthesis of highly complex natural products, pharmaceuticals, and functional materials.

## Graphical abstract

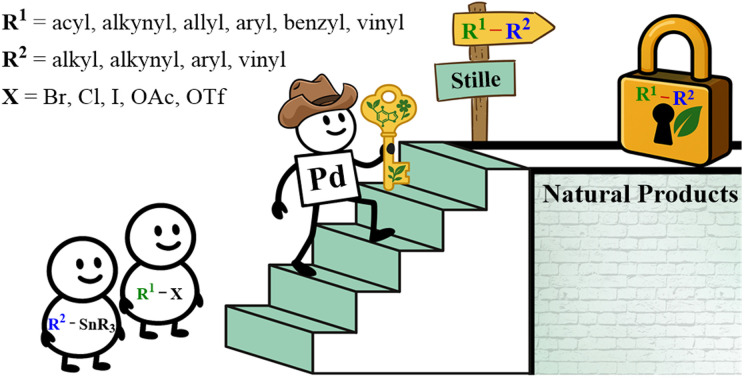

## 1. Introduction

Cross-coupling reactions are among the most fundamental tools in modern synthetic chemistry, enabling the precise construction of key molecular frameworks found in natural products, polymers, and bioactive compounds.^1^ The ability to form carbon–carbon (C–C) bonds is a cornerstone of organic synthesis and lies at the heart of strategies used to build natural product scaffolds and complex molecular architectures.^2,3^ These reactions have drawn increasing attention due to their mild and versatile conditions, made possible by the use of active ligands and transition-metal complexes. Such catalytic systems allow the efficient creation of intricate structures relevant to materials science, pharmaceuticals, and agrochemicals.^4,5^ Among transition-metal-mediated transformations, palladium-catalyzed C–C couplings are especially important for their profound impact on molecular architecture and for enabling the efficient assembly of complex carbon frameworks.^6–8^ Their global significance was recognized in the 2010 Nobel Prize in Chemistry, awarded to Heck, Negishi, and Suzuki for pioneering palladium-catalyzed cross-coupling reactions.^5^ The first iron-catalyzed cross-coupling reaction was reported by Tamura and Kochi,^9^ leading to the key strategies such as Suzuki,^10,11^ Heck,^12,13^ Sonogashira,^14,15^ Negishi,^16,17^ Stille,^18–20^ Cadiot–Chodkiewicz,^21^ Castro–Stephens,^22^ Hiyama,^23^ Liebeskind–Srogl,^24^ and Kumada reactions,^25^ expanding the role of cross-coupling in organic synthesis. Given their broad applicability, numerous reviews have highlighted recent advances in these methodologies.^26–28^ Due to their unique properties, natural products often serve as core structures in drug development.^29^

Among these reactions, the Stille coupling—a palladium-catalyzed cross-coupling between organostannanes and organic electrophiles—stands out for its robustness, tolerance of diverse functional groups, and efficiency in forming C–C bonds under mild conditions. Originally introduced by Kosugi^30^ and later refined by John K. Stille,^18,20^ this reaction has evolved over nearly four decades into a highly reliable and versatile method for bond construction.^31^ The general reaction involves coupling an organic halide or pseudohalide (R_1_–X) with an organostannane (R_2_–SnR_3_) in the presence of a palladium catalyst to afford the coupled product (R_1_–R_2_), as shown in eqn (1).1
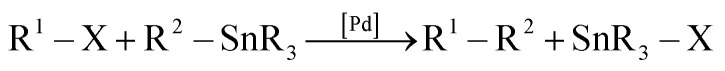


Its mild conditions, air and moisture stability, and high selectivity make it ideal for synthesizing complex molecules, including natural products and pharmaceuticals such as kinase inhibitors.^32,33^ Despite concerns over tin toxicity,^34^ innovations like bulky phosphines,^35^ bimetallic catalysis,^36–38^ and greener systems^39^ have sustained its relevance in modern organic synthesis.

**Scheme 1:**
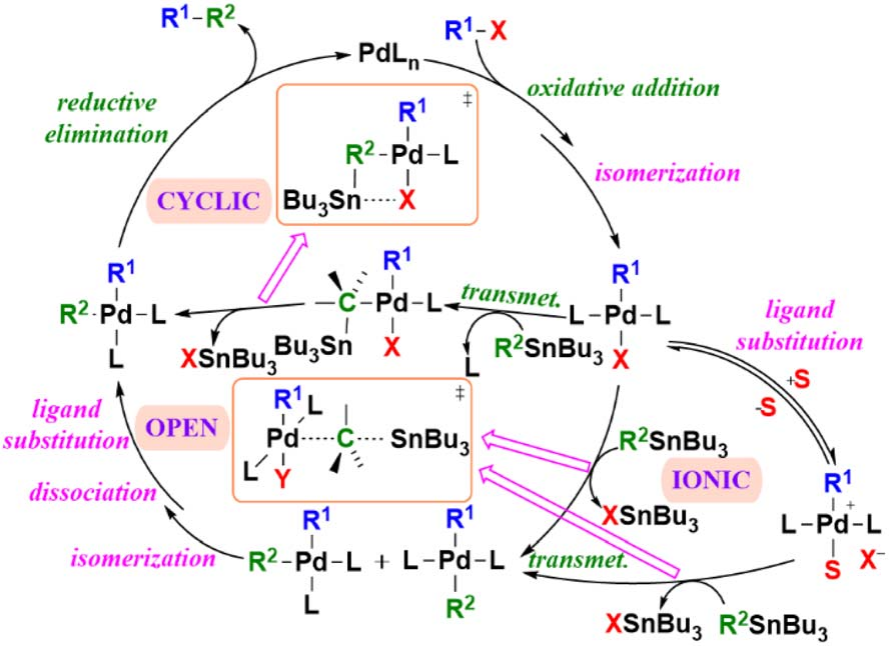
Pd-catalyzed Stille coupling mechanism.

Mechanistically, the Stille coupling involves a catalytic cycle consisting of oxidative addition, transmetalation, and reductive elimination, with possible participation of both cyclic and ionic pathways. *Espinet* and *Echavarren* provided detailed mechanistic studies highlighting the transmetalation step as the rate-determining and stereochemically sensitive process.^40^ This mechanistic complexity reflects the delicate interplay of ligands, solvents, and additives that govern product distribution and efficiency. The refined mechanistic model (Scheme 1) illustrates not only the canonical three-step pathway but also its dynamic, reversible intermediates that influence overall selectivity and yield.^41^

The Stille reaction remains a cornerstone of total synthesis, particularly in assembling complex natural products and functional materials. Building on foundational reviews,^42,43^ this work provides an updated overview of post-2018 developments and representative examples, highlighting both intermolecular and intramolecular variants that demonstrate the reaction's versatility, mechanistic diversity, and enduring significance in modern synthetic chemistry.

## 2. The intermolecular Stille coupling

**Scheme 2:**
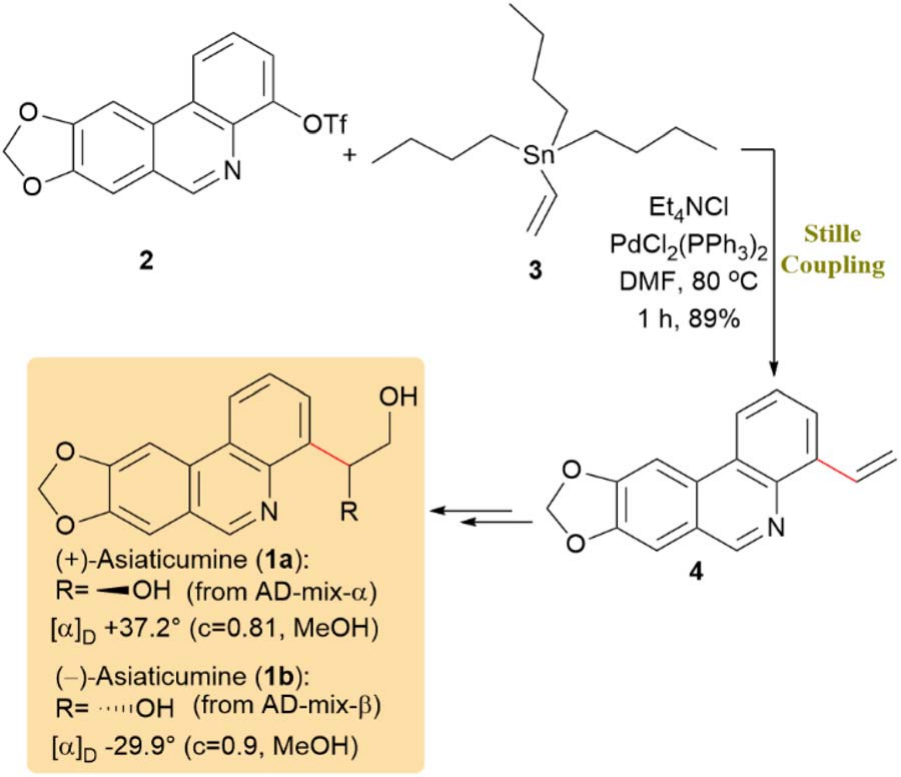
Coupling of triflate 2 with vinyltributyltin 3 formed 4-vinylphenanthridine (4), the key precursor to (+)-asiaticumine (1a) and (−)-asiaticumine (1b).

(+)-Asiaticumine A (1a) is a phenanthridine alkaloid with structural similarity to known bioactive natural products and is currently under evaluation for its biological activity. The initial synthesis of 1a and its enantiomer 1b was accomplished through the Stille reaction between triflate 2 and vinyltributyltin 3, employing PdCl_2_(PPh_3_)_2_ as the catalyst, resulting in the formation of 4-vinylphenanthridine (4) with an 89% yield. Compound 4 was subsequently transformed into 1a and its enantiomer 1b through Sharpless asymmetric dihydroxylation (Scheme 2).^44^

**Scheme 3:**
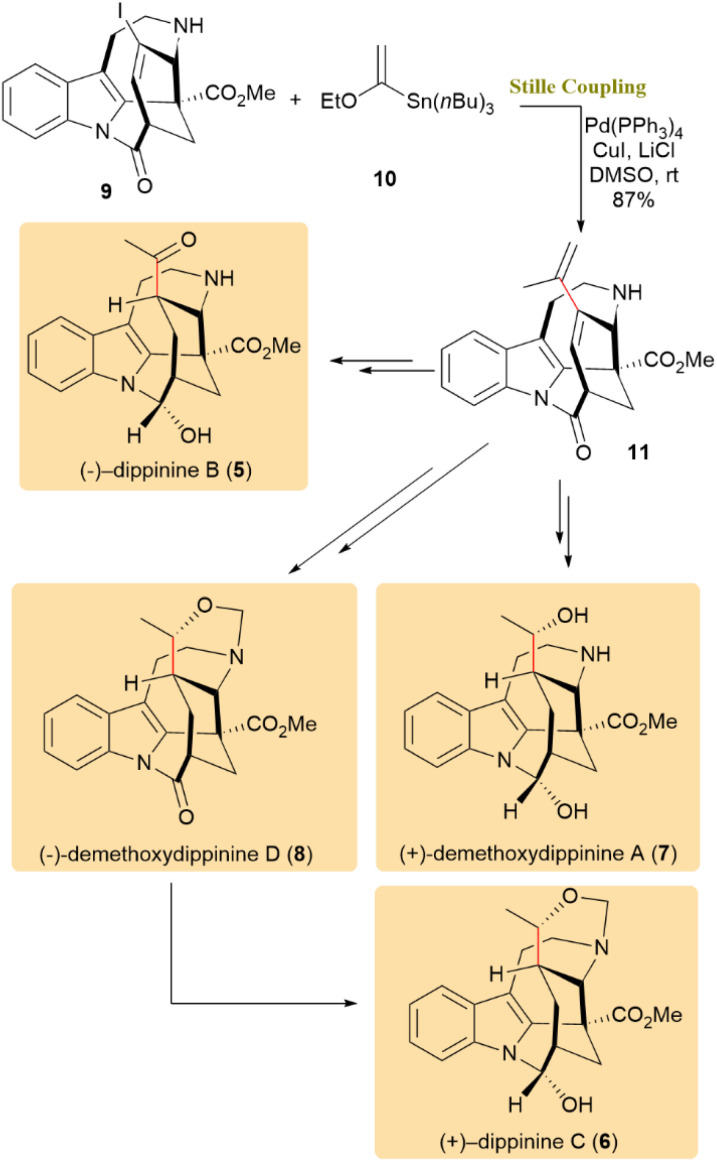
Stille coupling between 9 and ethoxyvinylstannane 10 to afford diene 11, which was elaborated into dippinine B (5), dippinine C (6), 11-demethoxydippinine A (7), and D (8).

The enantioselective total synthesis of the natural post-iboga indole alkaloids dippinine B (5) and C (6), along with analogues 11-demethoxydippinine A (7) and D (8), was initially reported by Han and colleagues.^45^ The synthesis involved the Stille cross-coupling of compound 9 with ethoxyvinylstannane 10, resulting in diene 11 with an 87% yield. This diene was then further processed to obtain the target alkaloids 5, 6, 7, and 8, which are of particular interest due to their unique structures and potential anticancer activity, notably against vincristine-resistant cells (Scheme 3).^45^

**Scheme 4:**
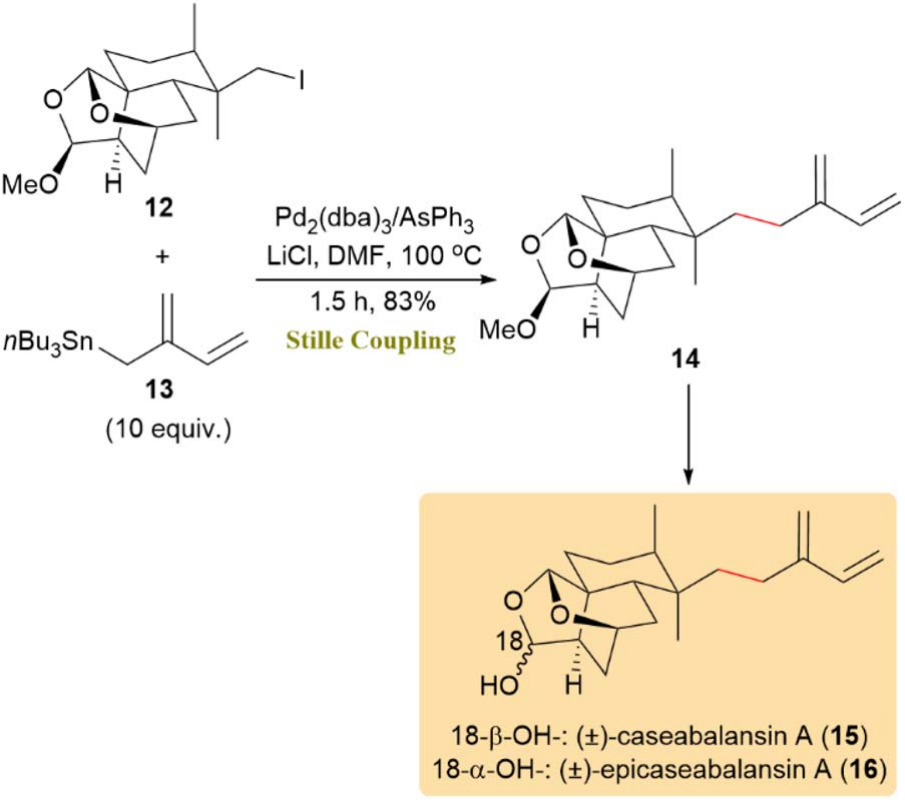
Coupling of alkyl iodide 12 with organostannane 13 gave compound 14, the intermediate which led to (±)-caseabalansin A (15) and (±)-18-epicaseabalansin A (16).

As illustrated in Scheme 4, a side chain added through the C(sp^3^)–C(sp^3^) Stille coupling of alkyl iodide 12 and organostannane 13, yielding the formation of key compound 14 under Pd_2_(dba)_3_/AsPh_3_ catalysis, which minimized the formation of undesired by-products. Compound 14 was then transfomed into (±)-caseabalansin A (15) and (±)-18-epicaseabalansin A (16), which are promising clerodane diterpenoids with selective cytotoxic activity against PC3 cancer cells.^46^

**Scheme 5:**
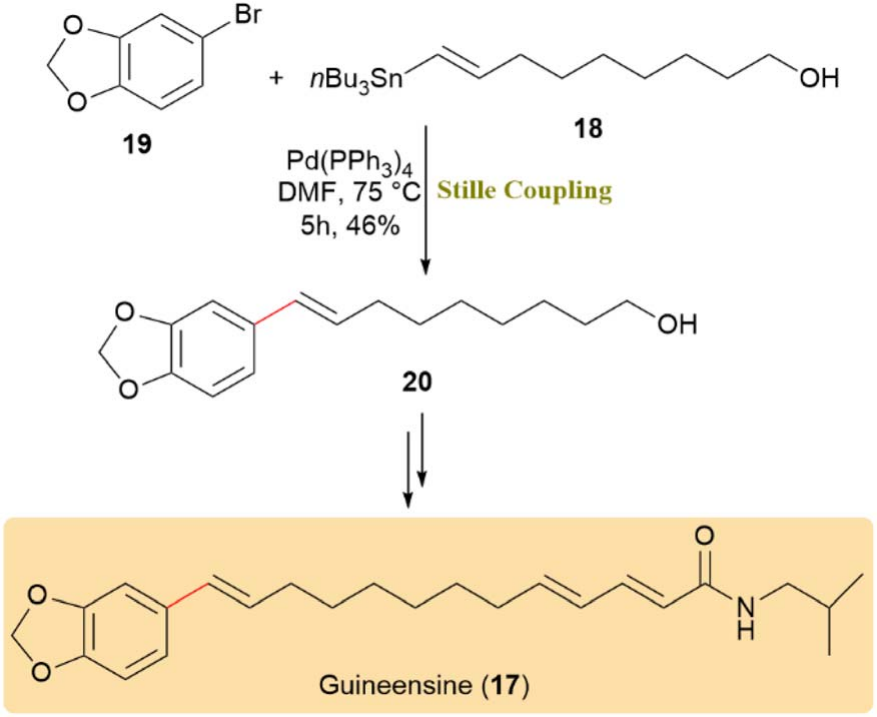
Stille reaction between stannane 18 and bromide 19 provided alcohol 20, a key intermediate en route to guineensine (17).

Guineensine (17), a plant-derived natural product, has gained attention for its powerful and selective inhibition of endocannabinoid uptake. It shows significant potential as a pharmacological tool and lead compound for the treating anxiety, pain, and inflammation. To obtain this bioactive molecule, a synthetic strategy based on cross-coupling was used. The synthesis involved the early introduction of the benzodioxole moiety through a Stille coupling between stannane 18 and bromide 19. This reaction yielded alcohol 20 in a 46% yield using Pd(PPh_3_)_4_ at 75 °C (Scheme 5).^47^

Heliolactone (21), a non-canonical strigolactone, exhibits promising biological activity by enhancing seed germination and inducing leaf senescence in both crops and parasitic weeds, making it a potential candidate for sustainable agricultural applications. Two independent total syntheses of heliolactone (21) have been reported, both of which involve a key Stille cross-coupling serving as the central bond-forming step.

**Scheme 6:**
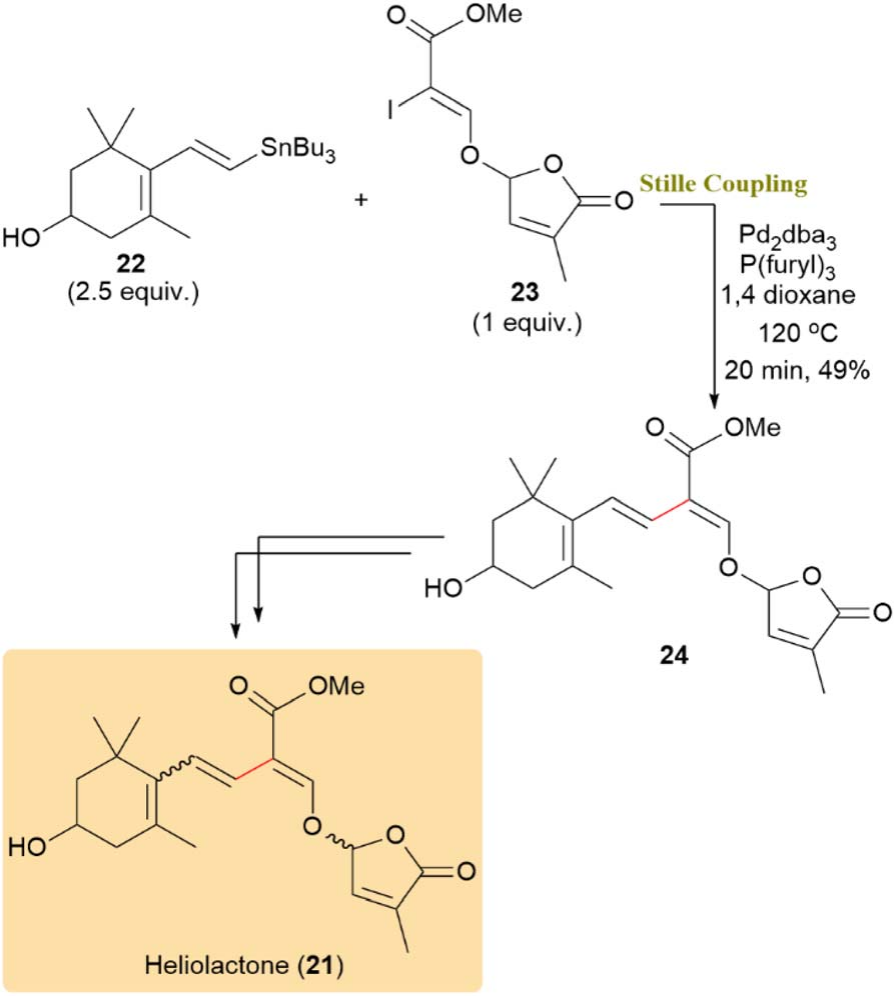
Coupling of dienyl stannane 22 with vinyl iodide 23 to afford compound 24, precursor to heliolactone (21).

In one strategy, a palladium-mediated cross-coupling was employed to couple dienyl stannane 22 and racemic vinyl iodide 23. Utilizing Pd_2_dba_3_ in combination with AsPh_3_, the transformation furnished the targeted coupling product in moderate yield. Screening of different catalytic systems demonstrated that the use of Pd_2_dba_3_ along with tris(2-furyl)phosphine, in the absence of any additive, delivered the most favorable result, affording compound 24 in a 49% isolated yield (Scheme 6).^48^

**Scheme 7:**
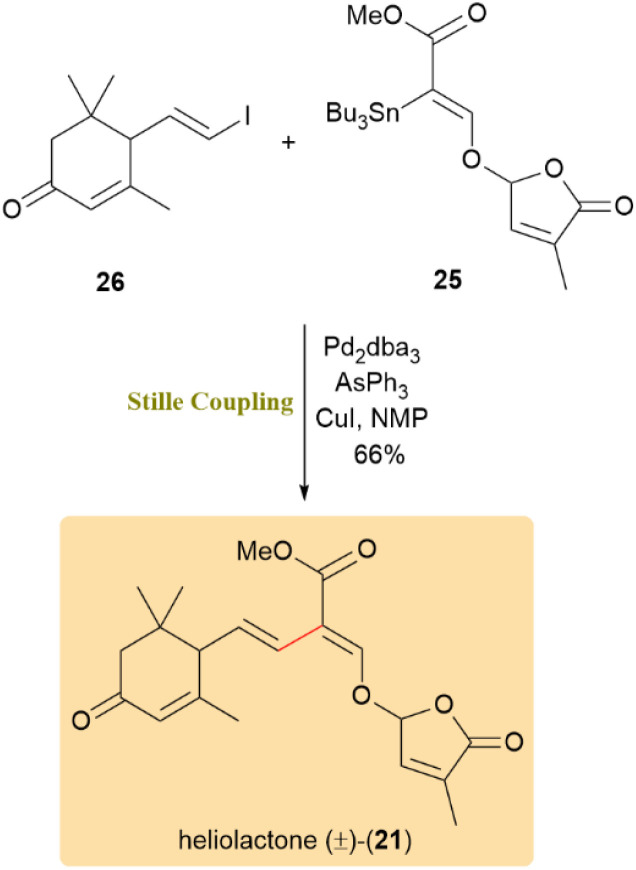
Reversed coupling of partners 25 and 26 delivered racemic heliolactone (±-21).

As shown in Scheme 7, The complete synthesis of heliolactone (21) was accomplished separately through a crucial Stille cross-coupling with reversed nucleophile–electrophile coupling partners. The optimization process involved preparing coupling partners 25 and 26, resulting in the racemic Heliolactone (±)-(21) being obtained at a yield of 66% under room temperature conditions.

**Scheme 8:**
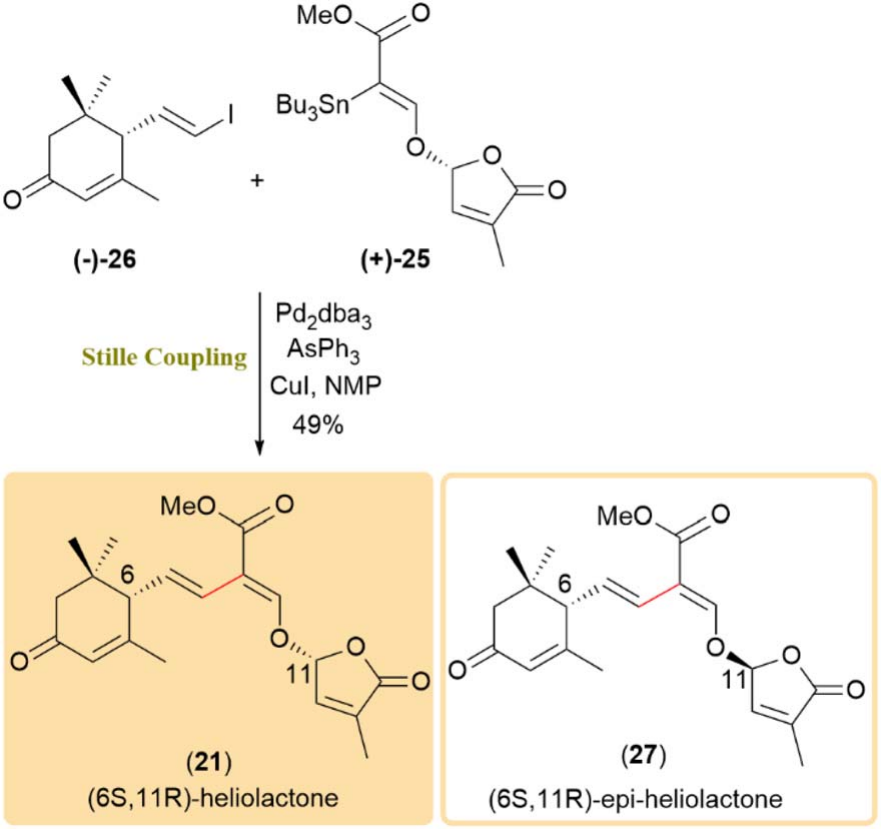
Enantioselective couplings of (−)-25/(+)-26 and (−)-25/(−)-26 produced heliolactone (21) and 11-*epi*-heliolactone (27).

Furthermore, enantioselective synthesis of heliolactone (21) was successful using (–)-25 and (+)-26, resulting in the formation of heliolactone. Additionally, coupling (–)-25 with (–)-26 produced 11-*epi*-heliolactone (27) similarly (Scheme 8).^49^

**Scheme 9:**
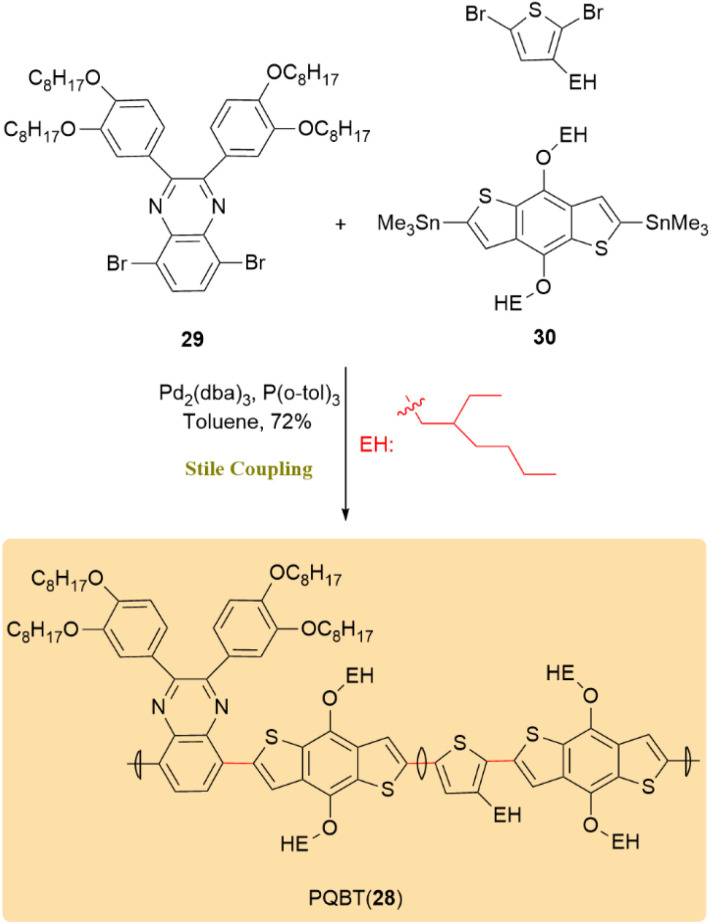
Coupling of quinoxaline 29 with benzodithiophene 30 to afford polymer 28 (PQBT).

The Stille coupling served as a critical step in assembling PQBT (28), a multifunctional polymer that exhibits ambipolar electrochromic behavior. This reaction facilitated the formation of the polymer backbone by linking quinoxaline 29 and benzodithiophene 30 units. Pd_2_(dba)_3_ in combination with P(*o*-tol)_3_ was employed as the catalytic system in toluene, resulting in a yield of 72% (Scheme 9). The mild reaction conditions, broad functional group compatibility, and efficiency of the Stille coupling were key factors in synthesizing a complex polymer with multifunctional properties.^50^

**Scheme 10:**
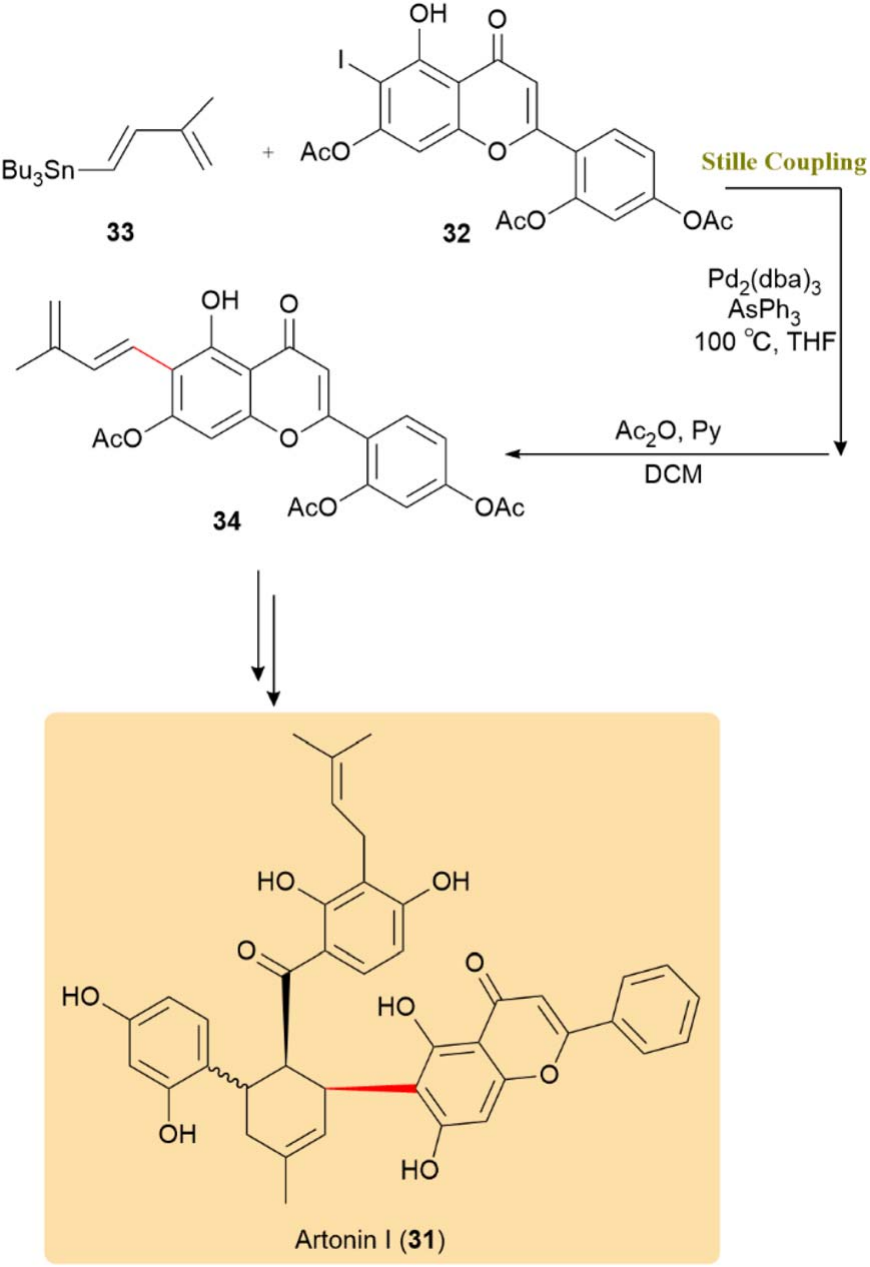
Iodide 32 couples with diene stannane 33 to form intermediate 34, enabling total synthesis of artonin I (31).

The reaction conditions for the Stille coupling, which was instrumental in the chemoenzymatic total synthesis of artonin I (31), were optimized to significantly increase yields from 5% to 75% by replacing Pd(OAc)_2_ with Pd_2_(dba)_3_ in the presence of AsPh_3_. In this reaction, iodide 32 and diene stannane 33 underwent coupling to afford diene precursor 34 (Scheme 10). This underscores the importance of catalyst choice and reaction conditions in organic synthesis and highlights the efficiency of the Stille reaction, particularly when integrated with enzymatic methods for natural product synthesis and drug discovery.^51^

**Scheme 11:**
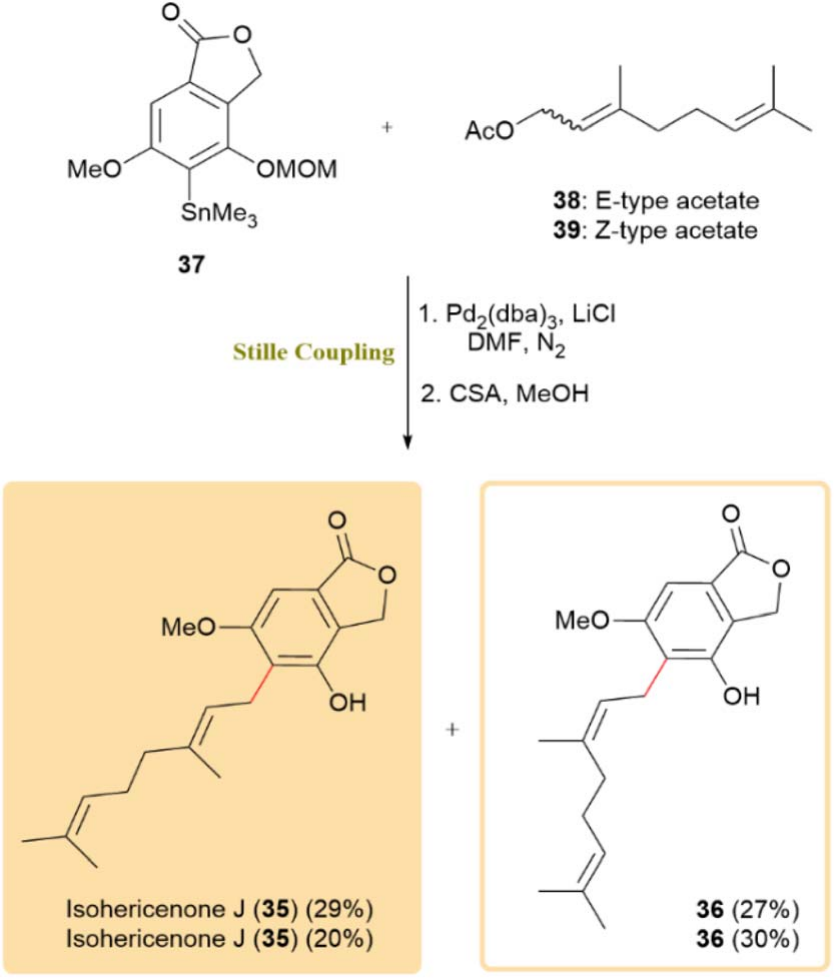
Affording isohericenone J (35) and its isomer (36) using Sille coupling.

**Scheme 12:**
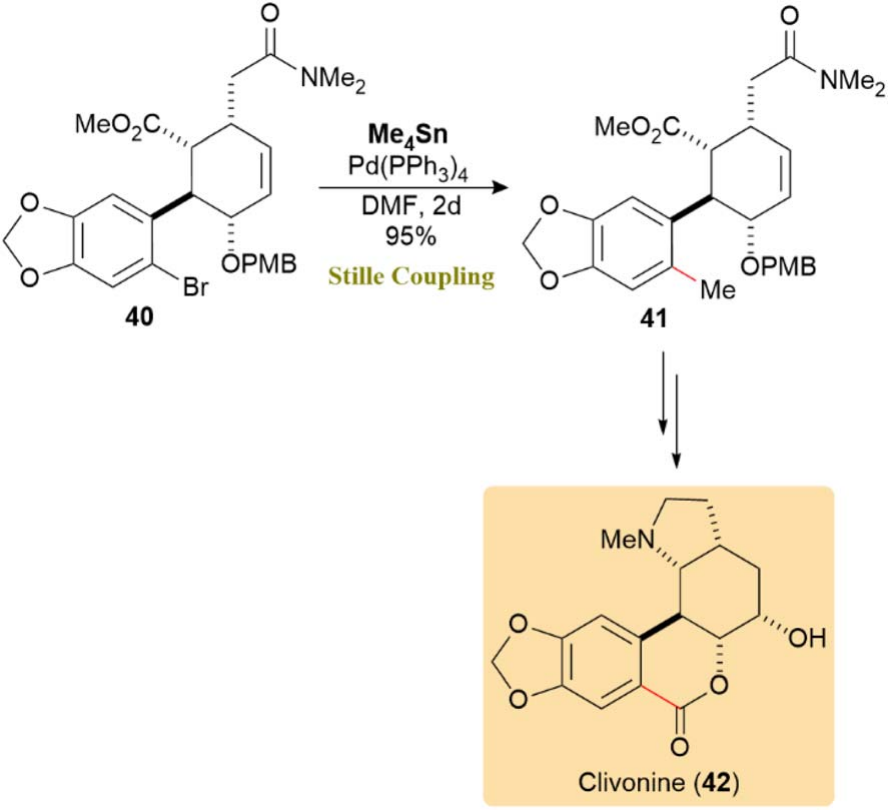
A Pd(PPh_3_)_4_-catalyzed Stille reaction of aryl bromide 40 gave product 41 and completed the synthesis of (±)-clivonine (42).

Isohericenone J (35), a cytotoxic metabolite isolated from *Hericium erinaceum*, along with its isomer (36), were synthesized through an optimized two-step sequence involving a Stille coupling followed by deprotection using d-camphorsulfonic acid, affording a combined yield of 29%. The critical Stille cross-coupling step was performed between a stannane intermediate bearing a lactone moiety (37) and (*E*)-3,7-dimethylocta-2,6-dien-1-yl acetate (38), enabling the formation of the C5–C1′ carbon–carbon linkage. Furthermore, employing (*Z*)-3,7-dimethylocta-2,6-dien-1-yl acetate (*Z*) under identical conditions generated a mixture of geometric isomers, yielding both *E*- and *Z*-products in a 2 : 3 ratio (Scheme 11).^52^

(±)-Clivonine, a lycorenine-type Amaryllidaceae alkaloid with significant anticancer, antimicrobial, and neuroprotective properties, serves as a valuable scaffold in medicinal chemistry. As shown in Scheme 12, the Stille reaction, facilitated by a Pd(PPh_3_)_4_ catalyst, exhibited high efficacy in coupling aryl bromide 40, resulting in product 41 with an impressive 95% yield over a 2-day period. This successful application highlights the versatility of the Stille reaction in forming aryl–aryl bonds, domenstrating its key involvement in assembling natural products like (±)-clivonine (42).^53^

**Scheme 13:**
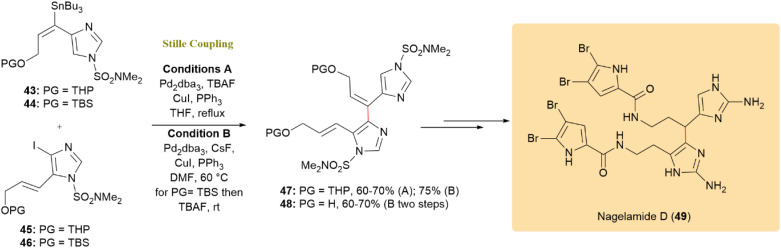
Vinylstannanes 43 and 44 coupled with iodoimidazoles 45 and 46 to form the bis-imidazolyl framework 47 leading to nagelamide D (49).

The nagelamides, a class of dimeric alkaloids from marine sponges, were synthesized through a Stille cross-coupling between vinylstannanes (43, 44) with iodoimidazoles (45, 46) using *Baldwin*'s modified conditions. This method, which CuI in combination with fluoride additives as co-catalytic agents, proved effective for sterically and electronically demanding substrates. Efficient cross-coupling was achieved under optimized conditions employing either CuI/TBAF in THF or CuI/CsF in DMF to produce the bis imidazolyl framework (47). Subsequent transformations resulted in the key intermediate 48 and ultimately led to the total synthesis of nagelamide D (49). CsF-mediated protocols consistently provided a 75% yield on scale-up, while TB AF-mediated yields were lower (Scheme 13).^54^

**Scheme 14:**
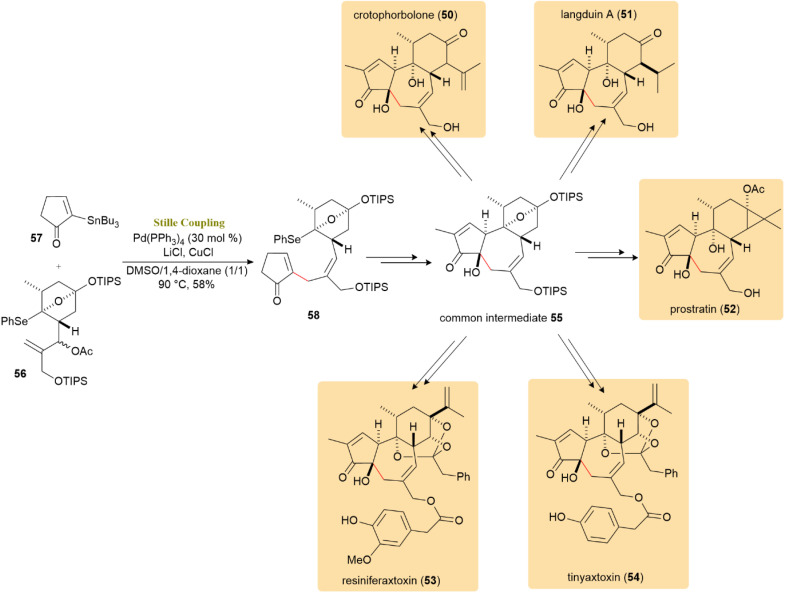
A π-allyl Stille coupling between 56 and 57 generated *E*-olefin 58, the key intermediate for crotophorbolone (50) and related diterpenoids.

A novel, unified strategy was devised for the efficient total synthesis of five prominent compounds across the rhamnofolane, tigliane, and daphnane diterpenoids families: crotophorbolone (50), langduin A (51), prostratin (52), resiniferatoxin (53), and tinyatoxin (54). Intermediate 55, with six stereocenters, was synthesized *via* a π-allyl variant of the Stille cross-coupling and then diversified into compounds 1–5 by selectively installing various functional groups. Treatment of compound 56 with vinyl tributylstannane 57, along with CuCl, LiCl and catalytic Pd(PPh_3_)_4_ in DMSO/1,4-dioxane at 90 °C, provided the target trisubstituted *E*-olefin, furnishing compound 58 as a stereochemically pure product in 58% yield (Scheme 14).^55^

**Scheme 15:**
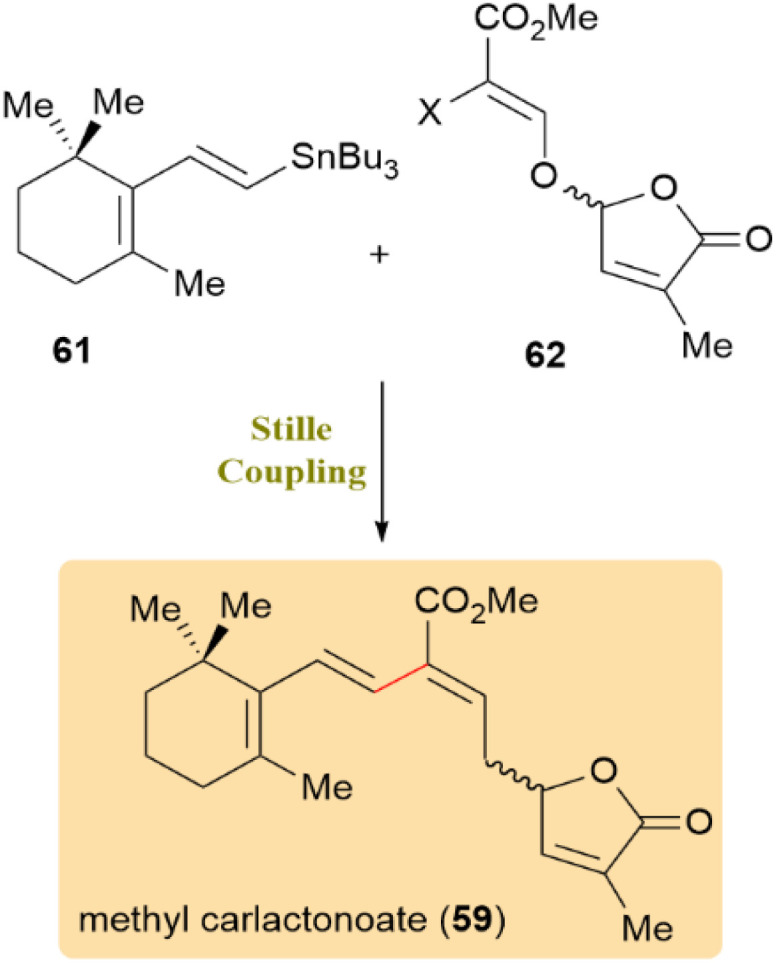
Vinyl stannane 61 and vinyl iodide 62 coupled to produce methyl carlactonoate (59).

A concise and effective route to enantiopure noncanonical strigolactones was established through a Stille coupling approach, which was achieved by synthesizing of methyl carlactonoate (59) and carlactonic acid (60). As shown in Scheme 15, this method involved the coupling of vinyl stannane 61 and vinyl iodide 62*via* the Stille reaction to synthesize methyl carlactonoate (59). Optimization of catalytic systems revealed Pd_2_dba_3_/AsPh_3_ as the most effective, yielding 75% of the desired product (Table 1).

**Table 1:** Optimization of palladium catalysts and reaction conditions for the Stille coupling of vinyl stannane 61 and vinyl iodide 62 in the synthesis of methyl carlactonoate (59)

No.	Catalyst (mol%)/halide	Additive/solvent	Temp (°C), time (min)	Conv. (%)	Yield (%)
1	Pd(PPh_3_)_4_ (20)/I	—/Toluene	100, 60	∼5%	Trace
2	PdCl_2_(PPh_3_)_2_ (20)/I	CuI/DMF	100, 60	100%	10
3	Pd_2_(dba)_3_ (10) + AsPh_3_ (40)/I	—/Dioxane	100, 60	100%	30
4	Pd_2_(dba)_3_ (7.5) + AsPh_3_ (30)/I	—/Dioxane	90, 45	100%	75
5	Pd_2_(dba)_3_ (10) + AsPh_3_ (40)/Br	—/Dioxane	100, 60	30%	Trace

**Scheme 16:**
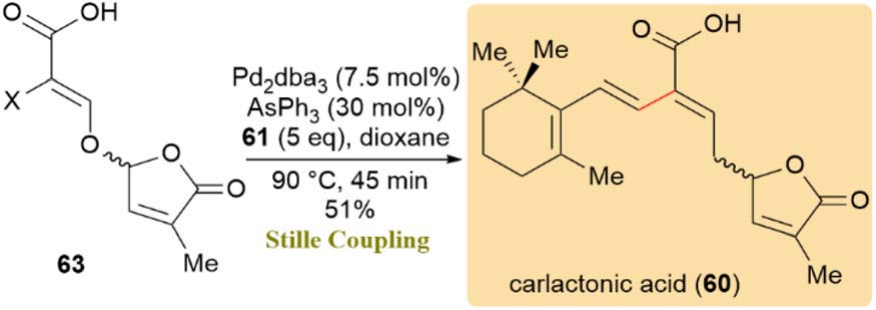
Carboxylic acid 63 and stannane 61 underwent Stille coupling to yield carlactonic acid (60).

Furthermore, the synthesis of carlactonic acid (60) was explored, resulting in a 51% yield *via* Stille cross-coupling between carboxylic acid 63 and a surplus of stannane 61 (Scheme 16). Notably, carlactonic acid (60) exhibited enhanced stability compared to methyl carlactonoate (59), remaining unchanged when maintained at −80 °C under inert argon for 30 days.^56^

**Scheme 17:**
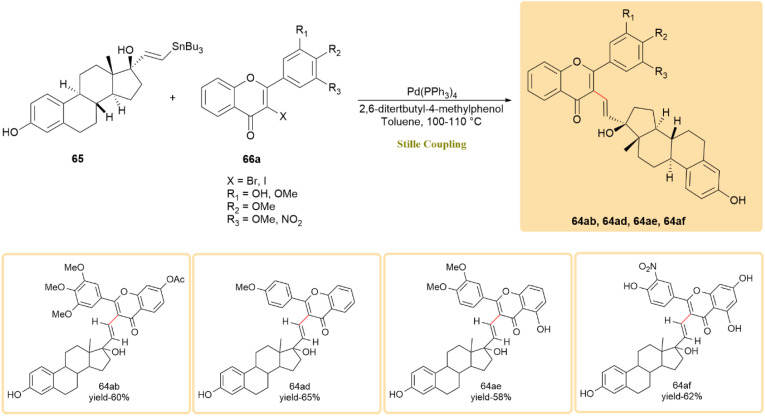
Tin estradiol derivative 65 and flavone 66a were coupled to form flavone-estradiol adducts 64ab–64af.

**Scheme 18:**
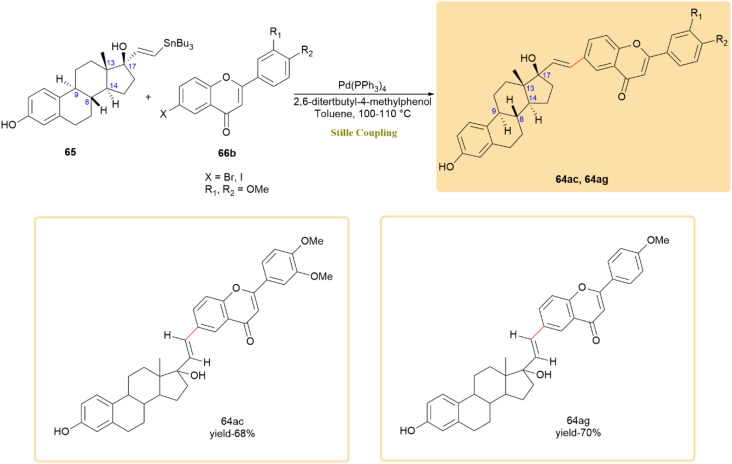
Tin estradiol 65 and flavone 66b underwent Stille reaction to produce flavone-estradiol adducts 64ab–64af.

Flavone–estradiol adducts (64ab–64af), which combine the estrogenic activity of estradiol with the anticancer properties of flavones, were synthesized *via* Still coupling.^57^ Tin estradiol derivatives 65 and flavone derivatives 66a (Scheme 17) and 66b (Scheme 18) reacted using a palladium catalyst and 3 crystals of 2,6-di-*tert*-butyl-4-methyl phenol in toluene at 100–110 °C, yielding products 64ab–64af in up to 70% yield within 2 days. These adducts were synthesized from natural 17β-estradiol and therefore retain its native stereochemistry (8*R*, 9*S*, 13*S*, 14*S*, 17*S*; 17β-OH), with no new chiral center formed at the coupling site.^58^

**Scheme 19:**
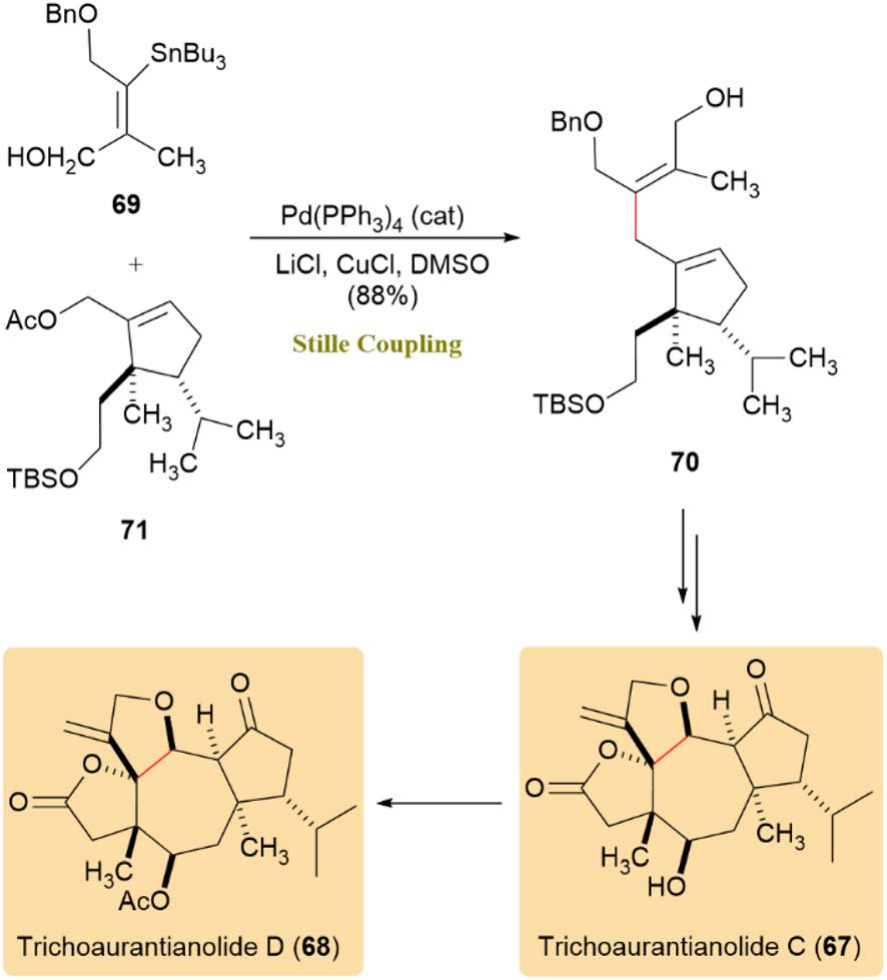
π-allyl Stille coupling to yield trichoaurantianolides C (67) and D (68).

A complete synthetic route to trichoaurantianolides C (67) and D (68), diterpenoid natural products isolated from tricholoma aurantium and tricholoma fracticum, was investigated. Demonstrating excellent stereocontrol, the π-allyl variant of Stille coupling was applied to generate nonconjugated 1,4-skipped dienes from tri- and tetrasubstituted alkenes. The stannane 69 substrate showed high reactivity, producing tetrasubstituted alkene 70 with an 88% yield and retention of olefin geometry when combined with allylic acetate 71 (Scheme 19). These results indicated that the Stille cross-coupling is a preferred approach for synthesizing highly substituted, stereodefined 1,4-dienes.^59^

**Scheme 20:**
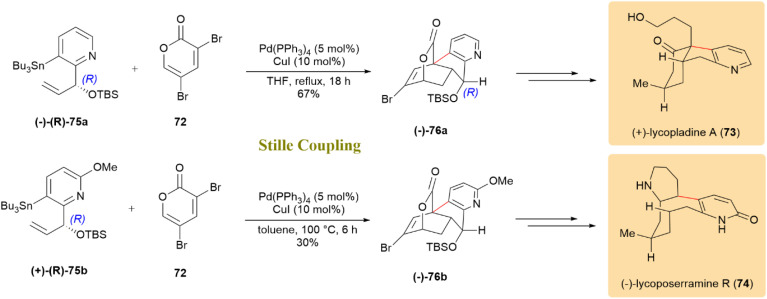
A tandem Stille/IMDA sequence from pyridine stannanes 75a, 75b to complete lycopladine A (73) and lycoposerramine R (74) synthesis.

As shown in Scheme 20, 2-pyrone derivative (72), bearing a chiral branched allylic silyl ether moiety, participated in an intramolecular Diels–Alder (IMDA) reaction, exhibiting excellent selectivity. This process yielded pure cycloadducts that were then transformed into (+)-lycopladine A (73) and (−)-lycoposerramine R (74). The tandem coupling/IMDA reaction involving pyridine stannane (−)-(*R*)-75a afforded tetracyclic lactone (−)-76a in a 67% yield, under reflux conditions with Pd(PPh_3_)_4_, successfully completing the total synthesis of 73. Subsequently, the synthesis of 74 was accomplished by employing pyridine stannane (+)-(*R*)-75b, leading to the formation of the IMDA adduct (−)-76b in a 30% yield with Pd(PPh_3_)_4_ serving as the catalyst at 100 °C.^60^

**Scheme 21:**
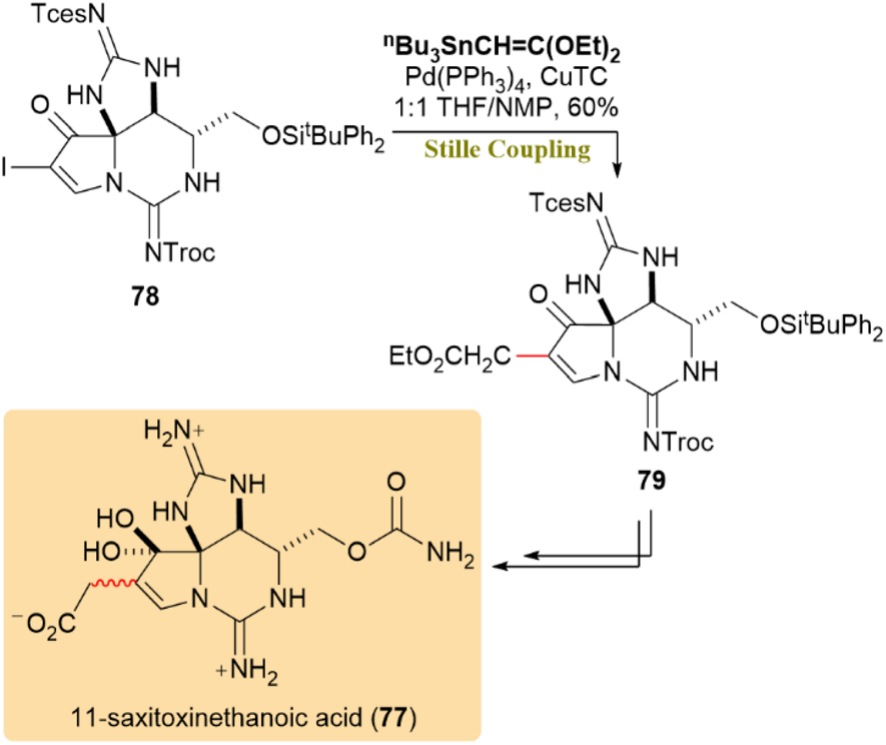
Vinyl iodide 78 and (2,2-diethoxyvinyl)tin coupled to form 79, a key precursor of 11-saxitoxinethanoic acid (77).

The Stille coupling was utilized to synthesize 11-saxitoxinethanoic acid (SEA) (77), a model compound for zetekitoxin AB, which is known for its potent inhibition of voltage-gated sodium channels (NaVs). Vinyl iodide 78 reacted with (2,2-diethoxyvinyl)tin employing Pd(PPh_3_)_4_ and CuTC, resulting in the formation of compound 79 with a 60% yield and consistent results (Scheme 21).^61^

**Scheme 22:**
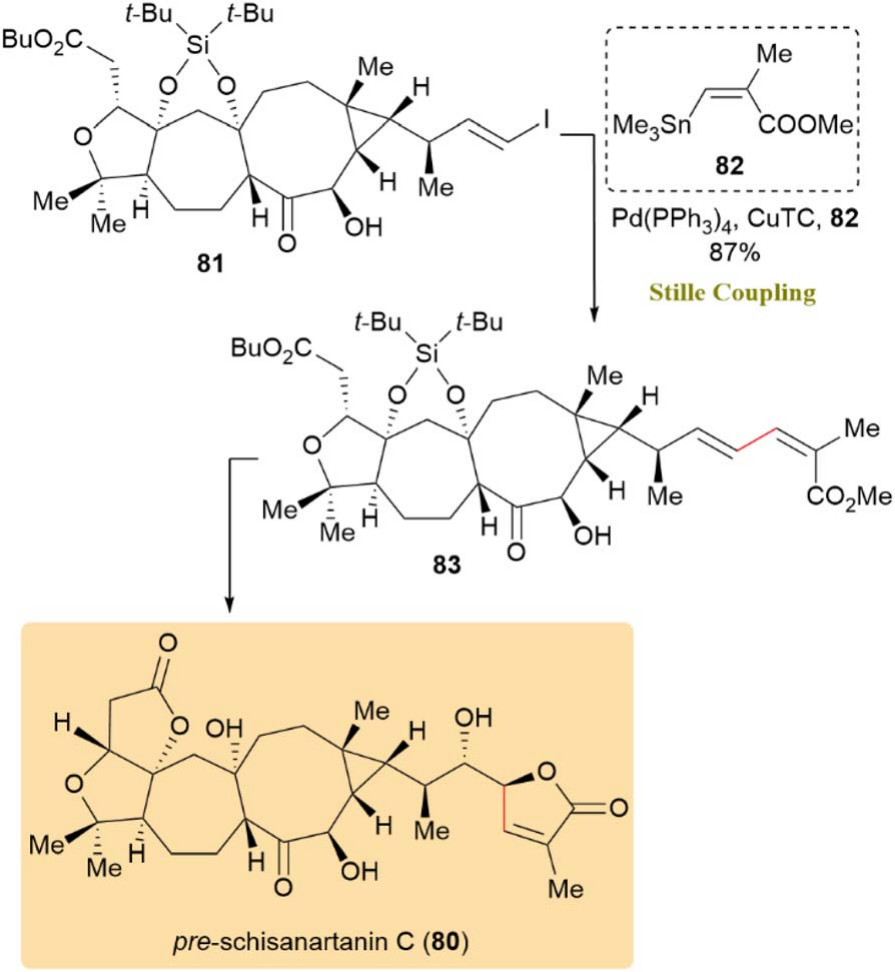
Produced 83 using Stille coupling, which was converted to pre-schisanartanin C (80).

The enantioselective total synthesis of pre-schisanartanin C (80), a Schisandra nortriterpenoid with significant antihepatitis, anti-tumor, and anti-HIV properties, has been reported. Vinyl iodide 81 underwent a Pd-catalyzed Stille reaction with trimethylstannylacrylate 82 and CuTC, yielding product 83 in an 87% yield. Further treatment of product 83 led to the isolation of pre-schisanartanin C (80) as the sole product (Scheme 22).^62^

**Scheme 23:**
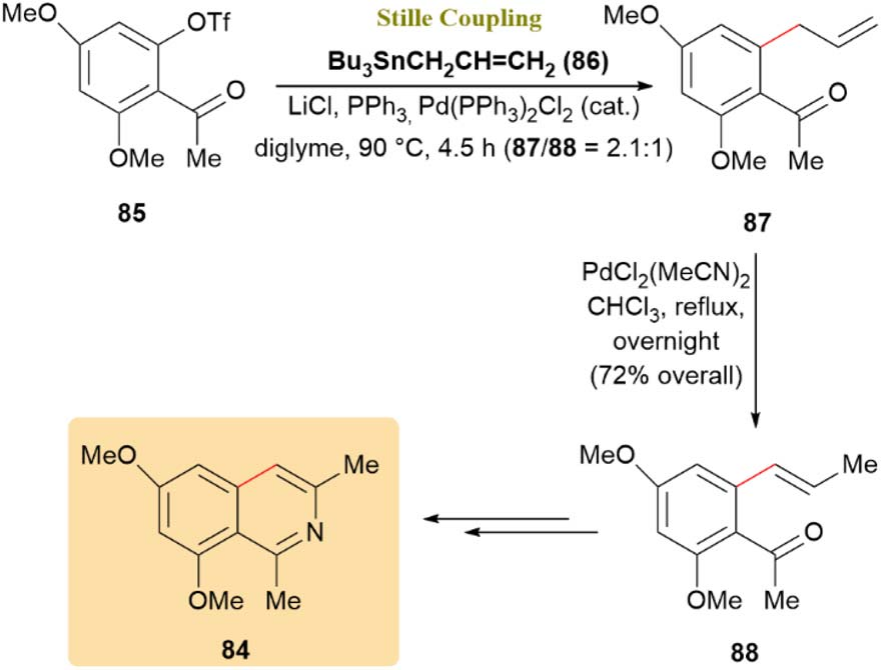
Completing the synthesis of 6,8-dimethoxy-1,3-dimethylisoquinoline (84).

The total synthesis of 6,8-dimethoxy-1,3-dimethylisoquinoline (84), a natural product from *Ancistrocladus tectorius*, along with evaluation of its cytotoxic activity, was investigated. A Pd(PPh_3_)_2_Cl_2_-mediated allylation of triflate 85 with allyltributyltin (86) in DMF at 100 °C afforded allyl 87 and propenyl 88 in a 2.1 : 1 ratio over 20 hours. This result was not unexpected, as the conversion of allyl benzenoids to 1-propenyl derivatives is common in Pd-catalyzed cross-couplings, particularly with electron-withdrawing substituents. Further treatment yielded 72% of 88, thereby achieving the synthesis of 84 (Scheme 23).^63^

**Scheme 24:**
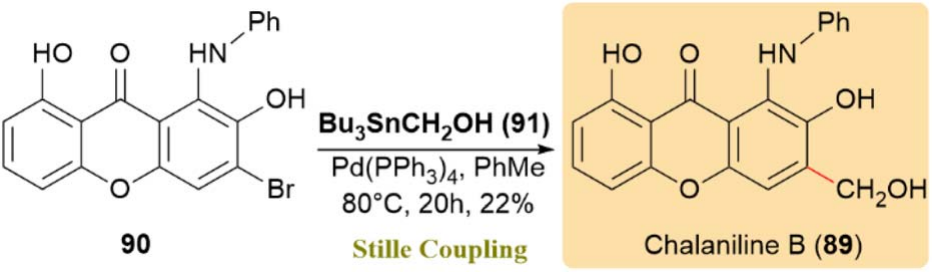
Chalaniline B (89) was formed from 90 through a palladium-catalyzed Stille coupling.

Chalaniline B (90), an antibiotic aminoxanthone, was synthesized from *Chalara* sp. treated with vorinostat. As shown in Scheme 24, compound 90 was converted directly into 89 through a palladium -catalyzed cross-coupling reaction using the unprotected variant of Migita's reagent (91), yielding 22%.^64^

**Scheme 25:**
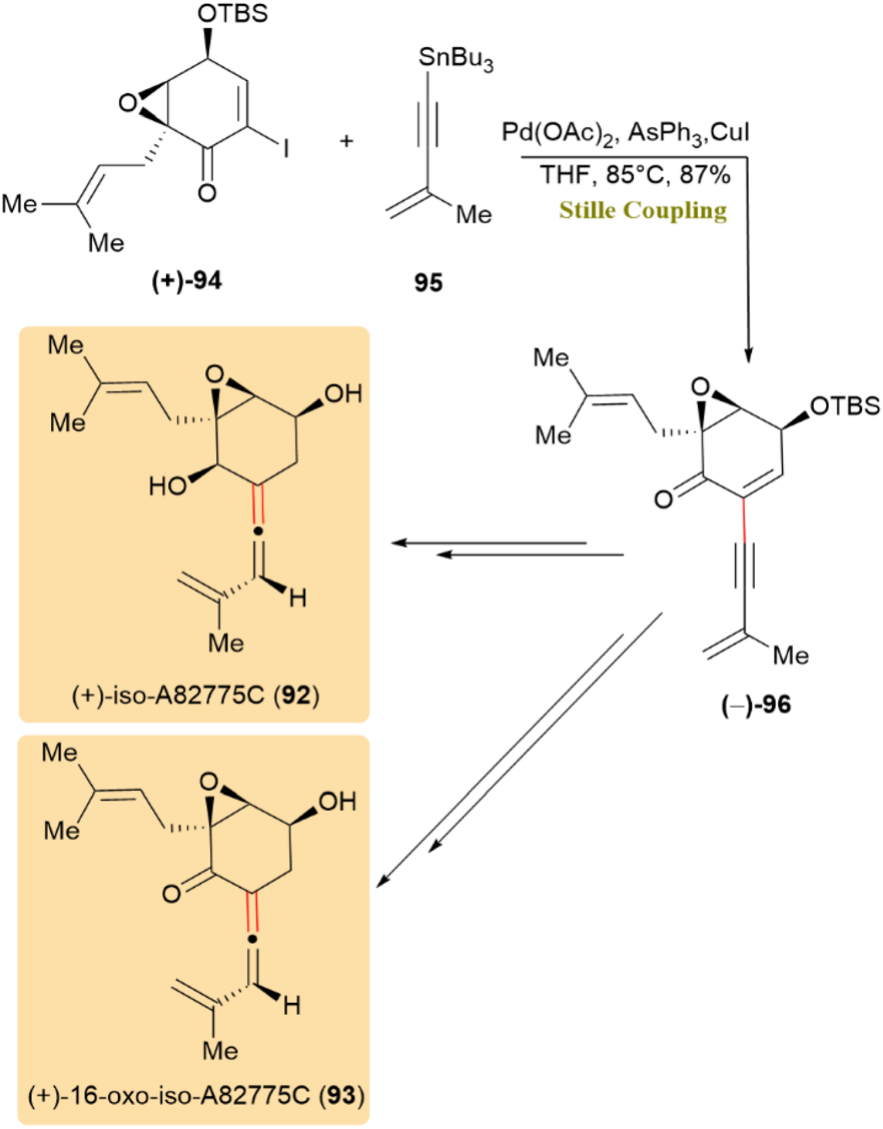
Iodide 94 and stannane 95 coupled to give alkyne 96, precursor of (+)-iso-A82775C (92) and (+)-16-oxo-iso-A82775C (93).

Epoxyquinoid natural products, which are a family of secondary metabolites with a complex cyclohexane core, exhibit antibiotic, antifungal, and antitumoral activities. The synthesis of (+)-iso-A82775C (92) and (+)-16-oxo-iso-A82775C (93) was achieved through Stille coupling. Iodide (+)-94 and organostannane 95 were coupled with an 87% yield using Pd(OAc)_2_, triphenylarsine, and copper(i) iodide, resulting in alkyne (−)-96, which was then further processed to obtain 92 and 93 (Scheme 25).^65^

**Scheme 26:**
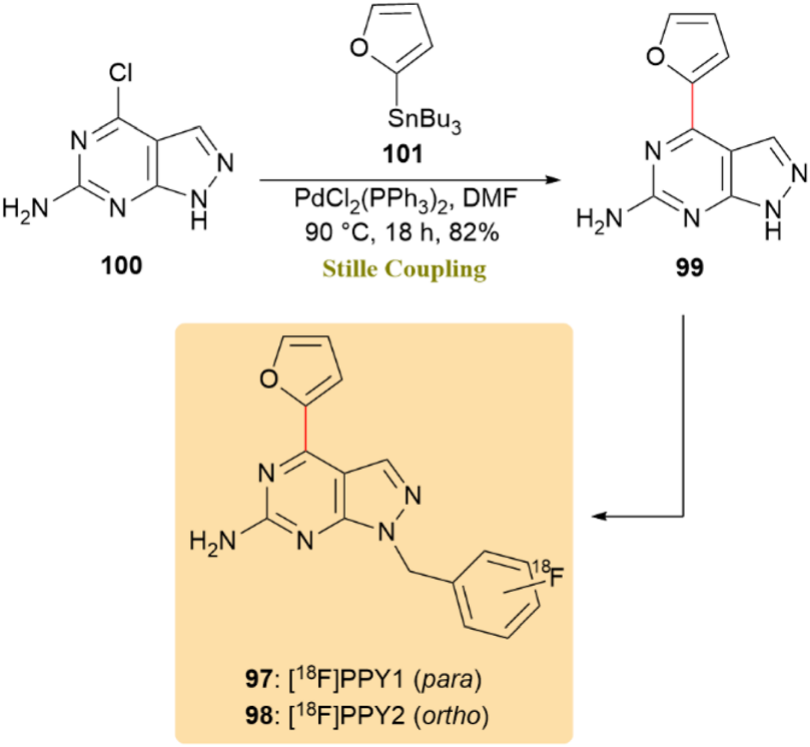
Stille coupling afforded 99, an intermediate for radiotracers [^18^F]PPY1 (97) and [^18^F]PPY2 (98).

A set of novel fluorinated pyrazolo[2,3-*d*]pyrimidine analogues (PPY1–PPY22) was prepared with the objective of developing an ^18^F-labeled PET radiotracer targeting the A_2_A adenosine receptor (A_2A_R) for brain imaging. This was accomplished by structural modification of a recently reported lead compound (PPY). The radiosynthesis of [^18^F]PPY1 (97) and [^18^F]PPY2 (98) was successfully performed using a newly developed alcohol-assisted, copper-mediated one-step ^18^F-labeling protocol. As shown in Scheme 26, compound 99 synthesized *via* stille coupling, served as a crucial intermediate for the development of subsequent derivatives. The optimization of the stille coupling process was achieved through the direct application of unprotected compound 100, resulting in a yield of 82% for compound 99 at 90 °C in the presence of tributyl(furan-2-yl)stannane 101.^66^

The total synthesis of (3*R*,4*S*)-4-hydroxylasiodiplodin (102), a bioactive resorcylic acid macrolactone with cytotoxic, antimicrobial, and prostaglandin biosynthesis inhibitory properties, was achieved using a Stille coupling as a pivotal step.

**Scheme 27:**
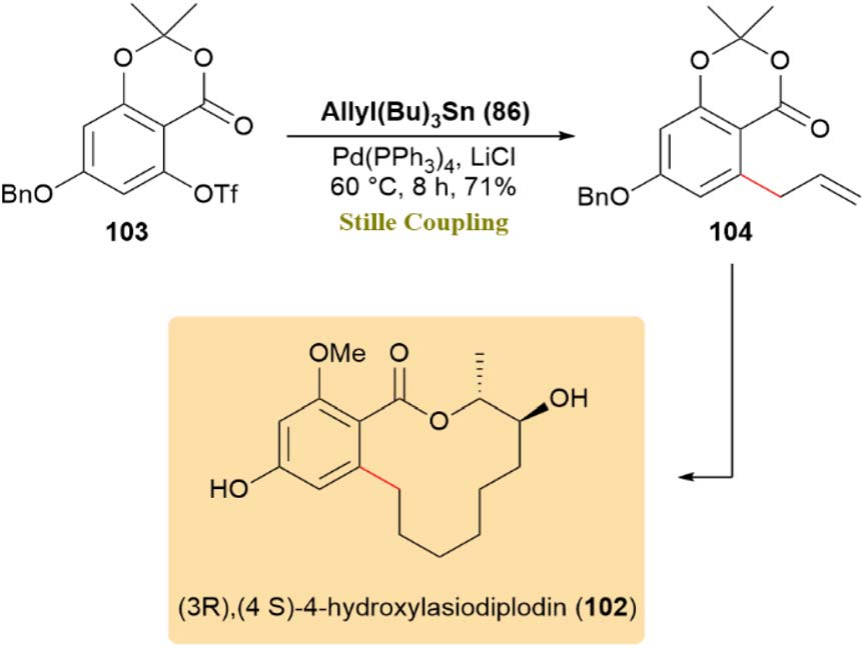
Triflate 103 and allylstannane 86 underwent Stille coupling to form 104, an intermediate toward (3*R*,4*S*)-4-hydroxylasiodiplodin (102).

**Scheme 28:**
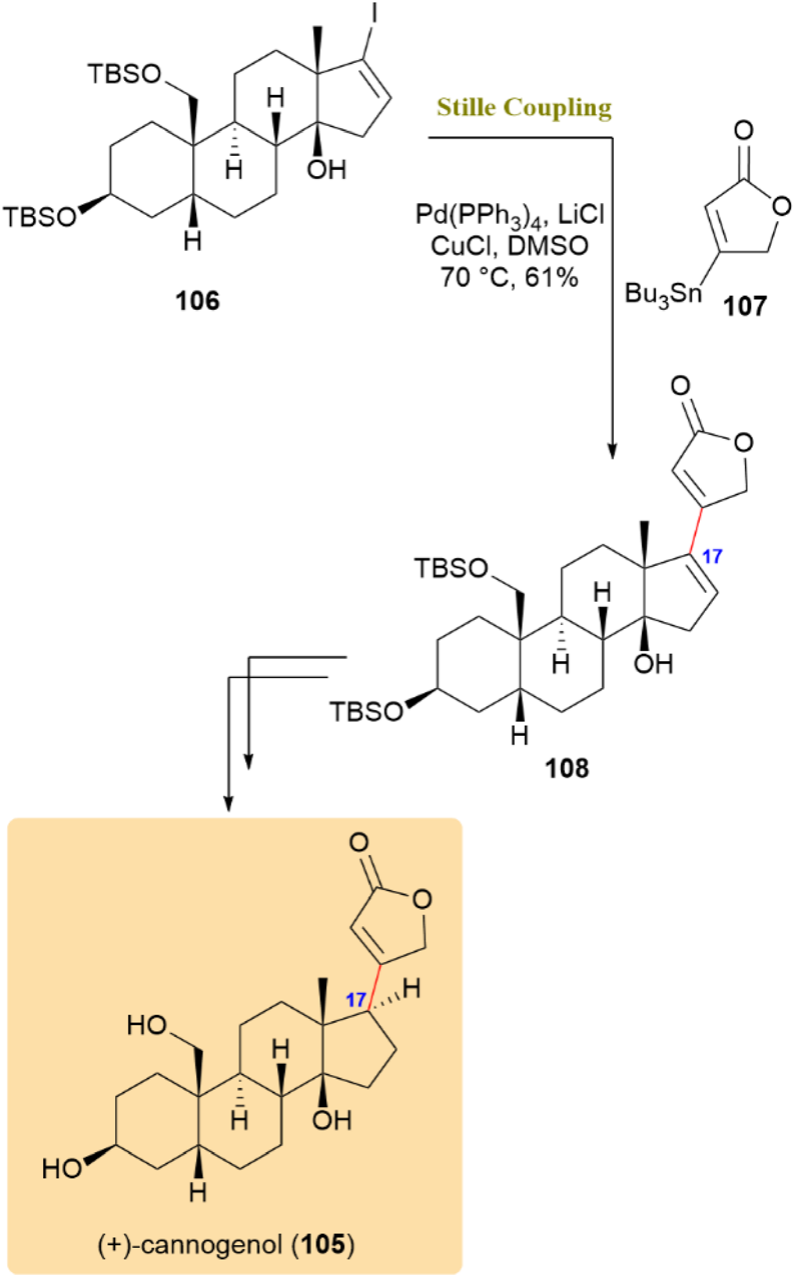
Stille coupling of compound 106 with tributylstannyl butenolide 107 serves as a key step in the synthesis of (+)-cannogenol (105).

The triflate compound 103 was utilized to form a C–C bond through palladium-catalyzed Stille coupling, employing allylstannane 86, LiCl, and Pd(PPh_3_)_4_, to afford compound 104 in 71% yield (Scheme 27). This intermediate facilitated the synthesis of compound 102.^67^

(+)-Cannogenol (105), an aglycone common to several biologically important cardiotonic glycosides, was synthesized through a key Stille coupling step. As shown in Scheme 28, the coupling of compound 106 with tributylstannyl butenolide 107 using Pd(PPh_3_)_4_, LiCl, and CuCl at 70 °C afforded compound 108 in 61% yield. This transformation efficiently installed the characteristic butenolide moiety at C17 of the steroid framework, a transformation that is challenging by other mSethods due to steric congestion and the sensitivity of the polyoxygenated D-ring. The LiCl/CuCl additives accelerated the transmetalation process and ensured clean coupling, highlighting the Stille reaction's superior tolerance toward complex functionalized steroid systems.^68^

**Scheme 29:**
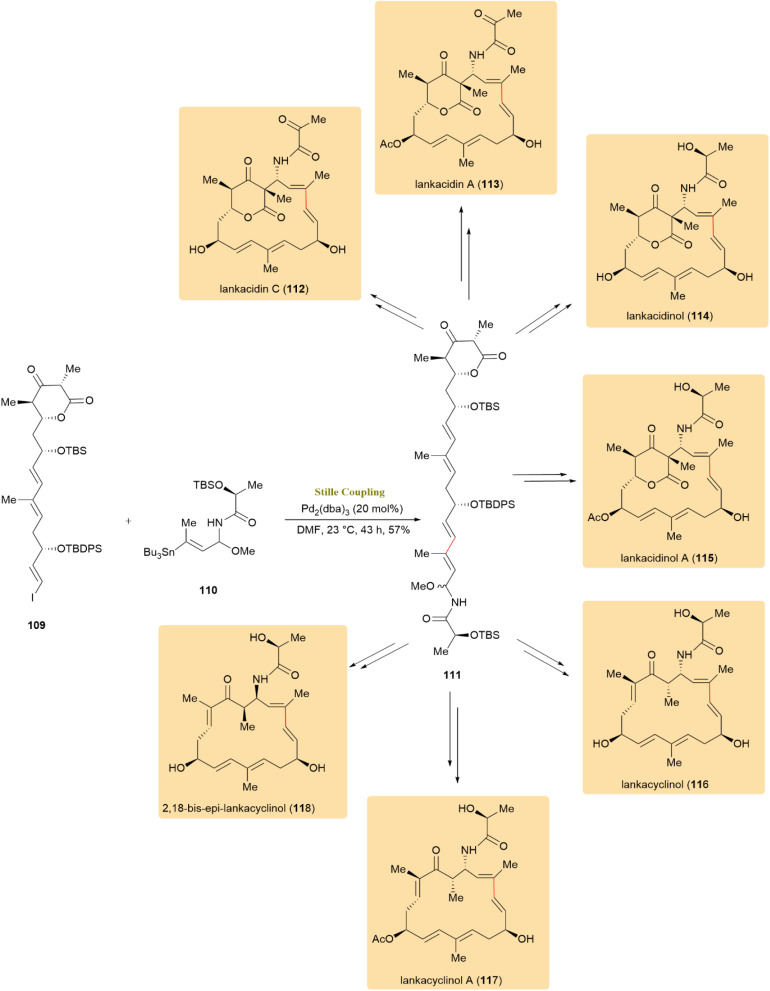
Compounds 109 and 110 coupled to give 111, converted into lankacidin derivatives 112–118.

Lankacidin antibiotics, known for their unique architectures and biological significance, have been extensively studied. The successful method involved the Stille reaction between compounds 109 and 110 under optimized conditions (20 mol% [Pd_2_(dba)_3_], DMF, 23 °C), yielding the hypersensitive *N*,*O*-acetal 111 with an isolated yield of 57%. Alternative methods proved unsuccessful, highlighting the crucial role of this reaction. Compound 111 was subsequently converted into various lankacidin derivatives, spincluding Lankacidin C (112), Lankacidin A (113), Lankacidinol (114), Lankacidinol A (115), Lankacyclinol (116), and Lankacyclinol A (117). Additionally, it was transformed into 2,18-bis-*epi*-lankacyclinol (118), a newly identified natural product, contributing to the understanding of lankacidin structural diversity (Scheme 29).^69^

**Scheme 30:**
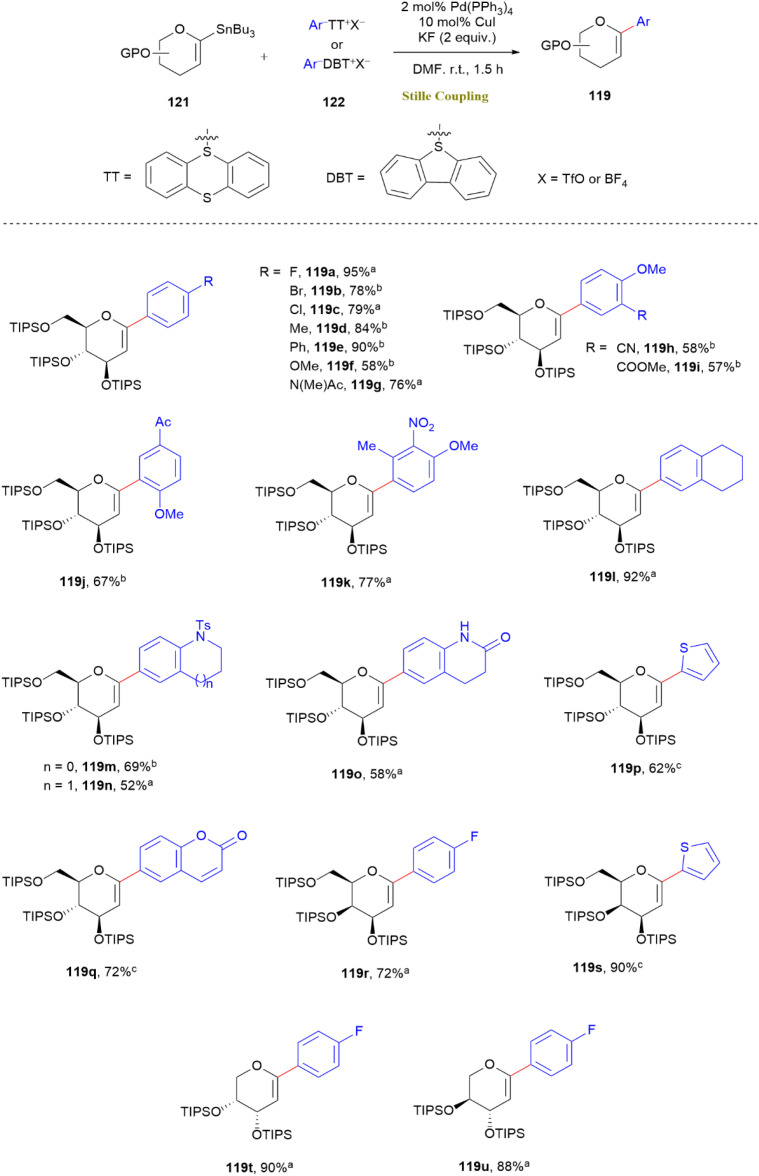
Synthesis of *C*-aryl glycals *via* Pd/Cu co-catalyzed coupling of glycosyl stannane 121 (0.1 mmol) and sulfonium salt 122 (0.12 mmol). Conditions: Pd(PPh_3_)_4_ (2 mol%), CuI (10 mol%), KF (0.2 mmol), DMF (1 mL), under N_2_. Yields are isolated. a = TT, TfO^−^; b = TT, BF_4_^−^; c = DBT, TfO^−^.

A highly efficient Pd/Cu-catalyzed Stille cross-coupling method has been devised for the preparation of *C*-ary (119-series) and *C*-alkenyl glycals (120-series) through the reaction of glycosyl stannanes (121) with aryl (122-series) and alkenyl (123-series) sulfonium salts. This methodology takes advantage of the synergistic roles of palladium and copper. Pd(0) undergoes oxidative addition with sulfonium salts, followed by CuI-facilitated transmetallation and reductive elimination. The reaction occurs under mild, scalable conditions without the need for prefunctionalized aryl halides. It demonstrates broad functional group tolerance, including cyano, ester, nitro, ketone, methoxy, and alkyl substituents (Scheme 30).

**Scheme 31:**
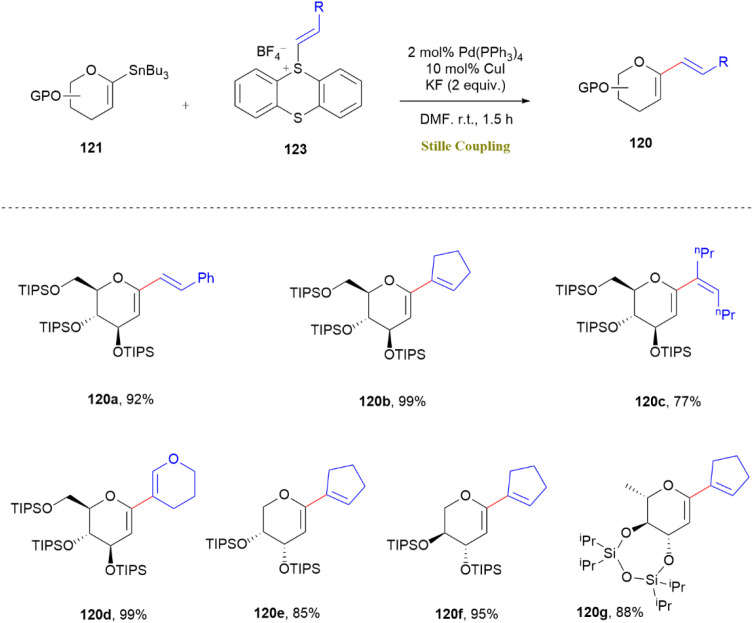
*C*-Alkenyl glycal synthesis *via* Pd/Cu-catalyzed coupling of stannane 121 (0.1 mmol) and sulfonium salt 123 (0.12 mmol) conditions: Pd(PPh_3_)_4_ (2 mol%), CuI (10 mol%), DMF (1 mL), KF (0.2 mmol), under N_2_. Yields are isolated.

Additionally, the strategy enabled one-pot formal C–H glycosylation, streamlining the incorporation of glycosylmoieties onto arenes. Notably, alkenyl sulfonium salts (123-series) exhibited higher reactivity than their aryl counterparts, resulting in alkenylated products (120a–120g) without *E*/*Z* isomerization (Scheme 31). This approach expanded the utility of the Stille reaction in carbohydrate chemistry, making it easier to synthesize of bioactive glycals relevant to drug discovery and natural product synthesis.^70^

**Scheme 32:**
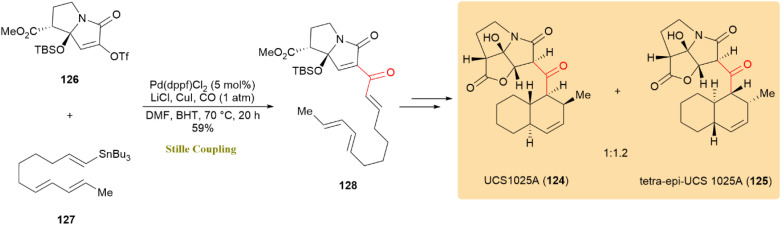
Vinyl triflate 126 and vinylstannane 127 underwent carbonylative Stille coupling to afford enone 128, a precursor of UCS1025A (124).

A convergent synthetic approach was established for the total synthesis of UCS1025A (124) and its diastereomer, tetra-*epi*-UCS1025A (125). This method employed a tandem carbonylative Stille coupling followed by a Diels–Alder cycloaddition to establish a critical carbon–carbon bond and assemble the *trans*-decalin core structure. UCS1025A (124) belongs to a class of natural compounds with antibacterial, antifungal, and anticancer activities. The carbonylative Stille cross coupling was applied to vinyl triflate 126 and vinylstannane 127, resulting in the smooth production of carbonylation enone 128 with Pd(dppf)Cl_2_ as a catalyst, achieving a 59% yield under specific conditions (Scheme 32).^71^

**Scheme 33:**
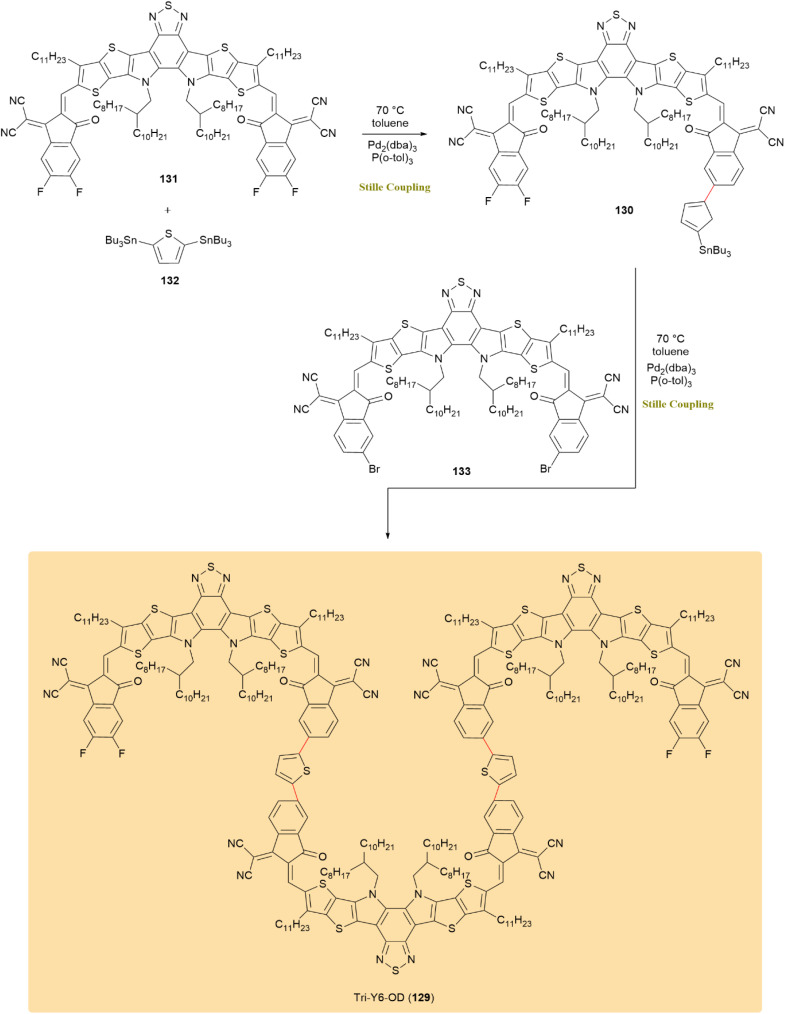
Monomer 131 and bis(stannyl)thiophene 132 coupled to form intermediate 130 for trimer acceptor tri-Y6-OD (129).

A stepwise method utilizing the Stille coupling was developed for synthesizing oligomer acceptors in organic-based solar cells. The successful synthesis of a trimer acceptor, Tri-Y6-OD (129), demonstrated the effectiveness of this approach, with oligomerization shown to improve both device performance and stability. The crucial intermediate 130 was obtained through a Stille coupling between a monobrominated monomer 131 with an excess 2,5-bis(tributylstannanyl)thiophene 132 mediated by Pd_2_(dba)_3_ in combination with P(*o*-tol)_3_. After purification by methanol precipitation and ethanol rinsing, the final product, Tri-Y6-OD 5, was obtained through a subsequent Stille coupling with dibrominated monomer 133 (Scheme 33).^72^

**Scheme 34:**
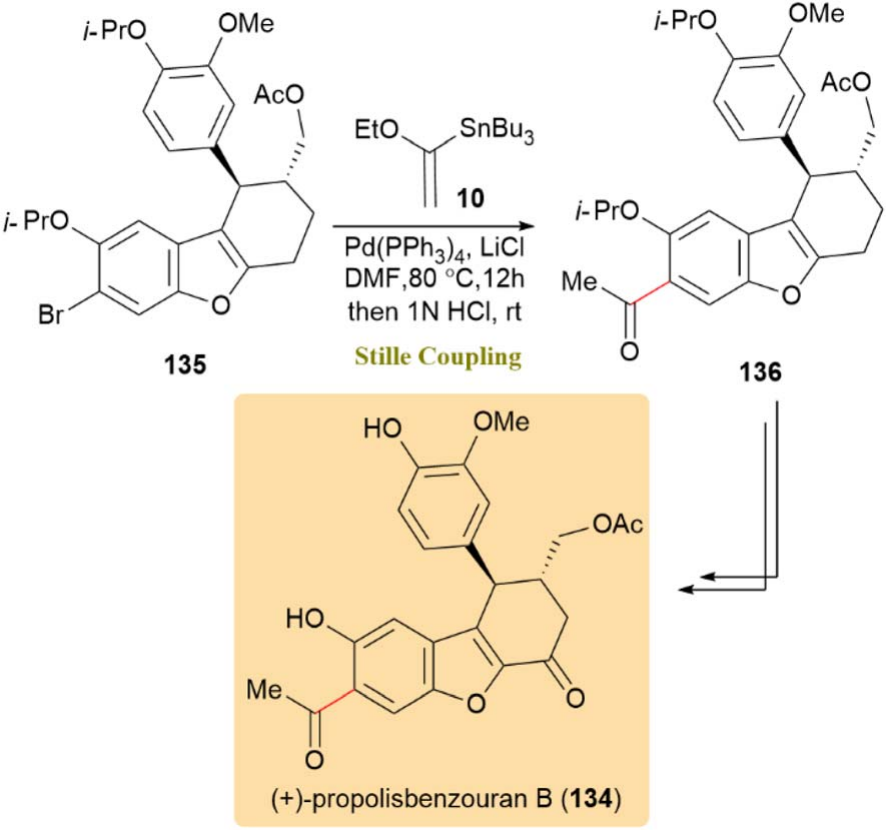
Stille coupling of tricyclic core 135 and (α-ethoxyvinyl)tributyltin 10 led to (+)-propolisbenzofuran B (134).

The first enantioselective synthesis of the cytotoxic natural compound (+)-propolisbenzofuran B (134) was completed in 11 synthetic steps. The key step involved a Pd(PPh_3_)_4_-catalyzed Stille coupling of the advanced tricyclic core 135 with (α-ethoxyvinyl)tributyltin 10, serving as a masked acetylating agent, in DMF at 80 °C. Acidic treatment afforded the intended C6-acetyl compound 136 with a yield of 84%. Subsequent treatment of this compound successfully produced 134 (Scheme 34).^73^

**Scheme 35:**
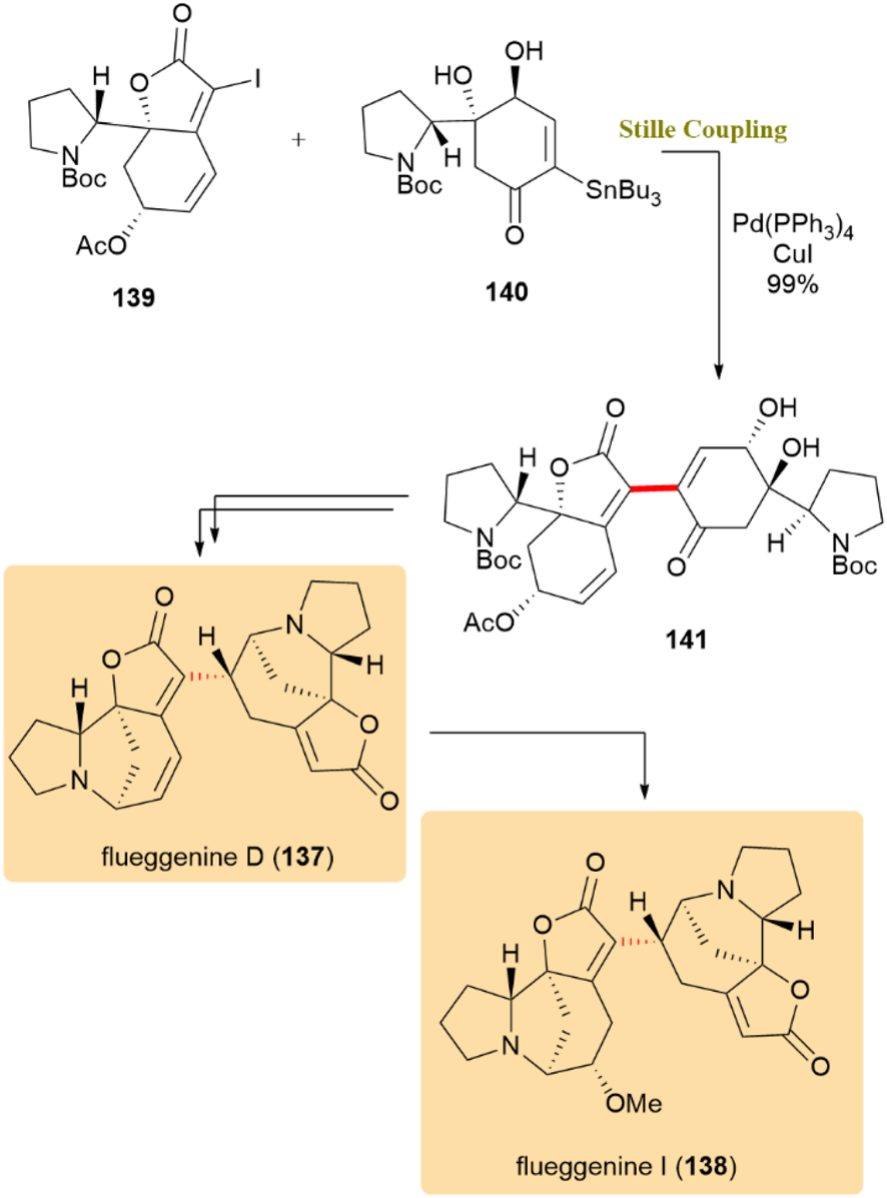
α-Iodobutenolide 139 and stannane 140 coupled to produce intermediate 141 toward flueggenines D (137) and I (138).

The total synthesis of flueggenines D (137) and I (138), representative dimeric Securinega alkaloids, was achieved through a key Stille coupling. This crucial step, which involved α-iodobutenolide 139 and stannane 140, employing Pd(PPh_3_)_4_ with CuI, yielded intermediate 141 in 99% yield (Scheme 35). These alkaloids are valuable models for exploring biosynthetic oligomerization pathways and for studying stereocontrolled conjugate reductions in natural product synthesis.^74^

**Scheme 36:**
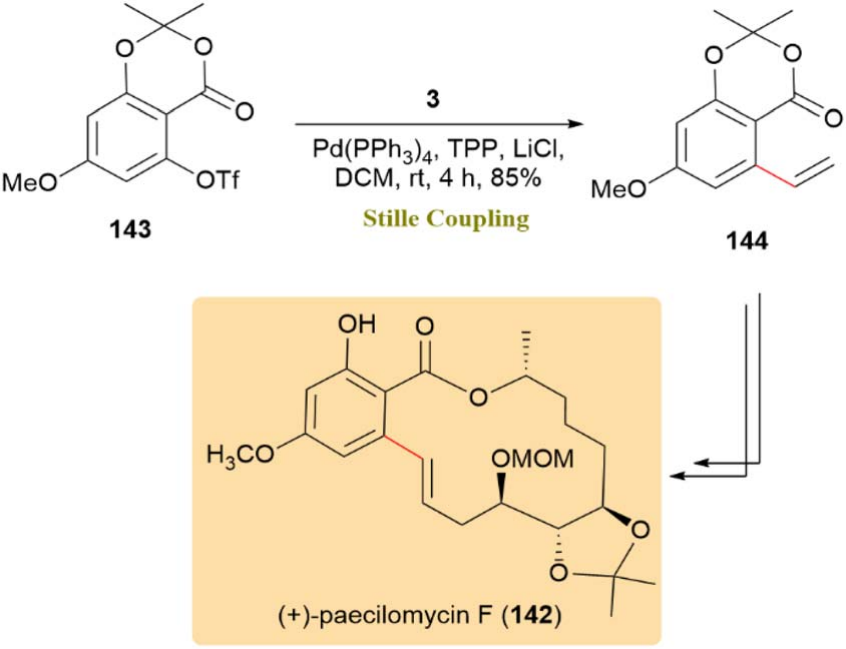
Triflate 143 and tributyl(vinyl)tin 3 coupled to afford vinylated ester 144, precursor of (+)-paecilomycin F (142).

The synthesis of protected (+)-paecilomycin F (142), a β-resorcylic acid lactone with significant antiplasmodial, antifungal, and antiviral activity, has been successful accomplished. A crucial step in the process included a Stille coupling of triflate 143 with tributyl(vinyl)tin (3) under Pd(PPh_3_)_4_, TPP, LiCl and DCM reaction conditions, leading to the creation of the vinylated aromatic ester 144 intermediate with an 85% yield (Scheme 36).^75^

**Scheme 37:**
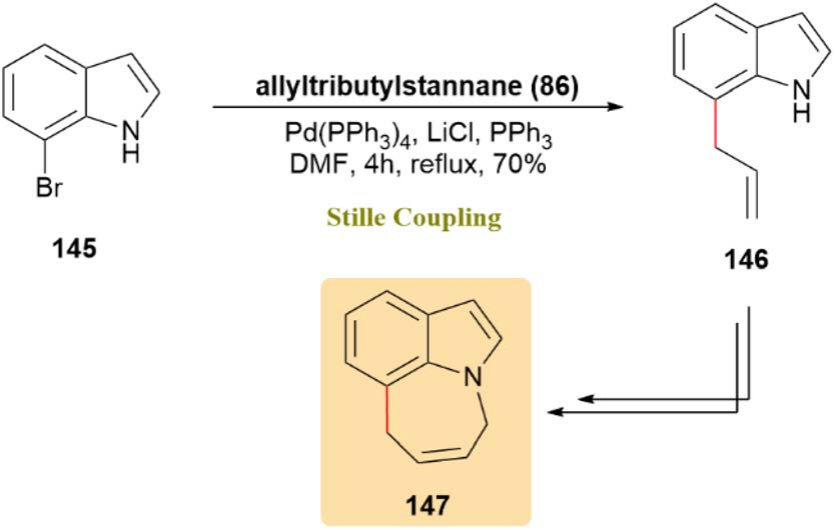
Synthesis of azepino[3,2,1-hi]indole (147) through Stille coupling.

Azepino[3,2,1-hi]indoles, which contain seven-membered ring tricyclic structures, have been recognized for their significance in medicinal chemistry. In an effort to develop new synthetic strategies for these compounds, commercially available 7-bromo-1*H*-indole (145) was treated with allyltributylstannane 86 employing a Pd(0) catalyst for cross-coupling, yielding the allyl derivative (146) with a 70% yield. Subsequent treatments of compound 146 resulted in the formation of azepino[3,2,1-hi]indole derivatives, including [3,2,1-hi]indole (147) (Scheme 37).^76^

**Scheme 38:**
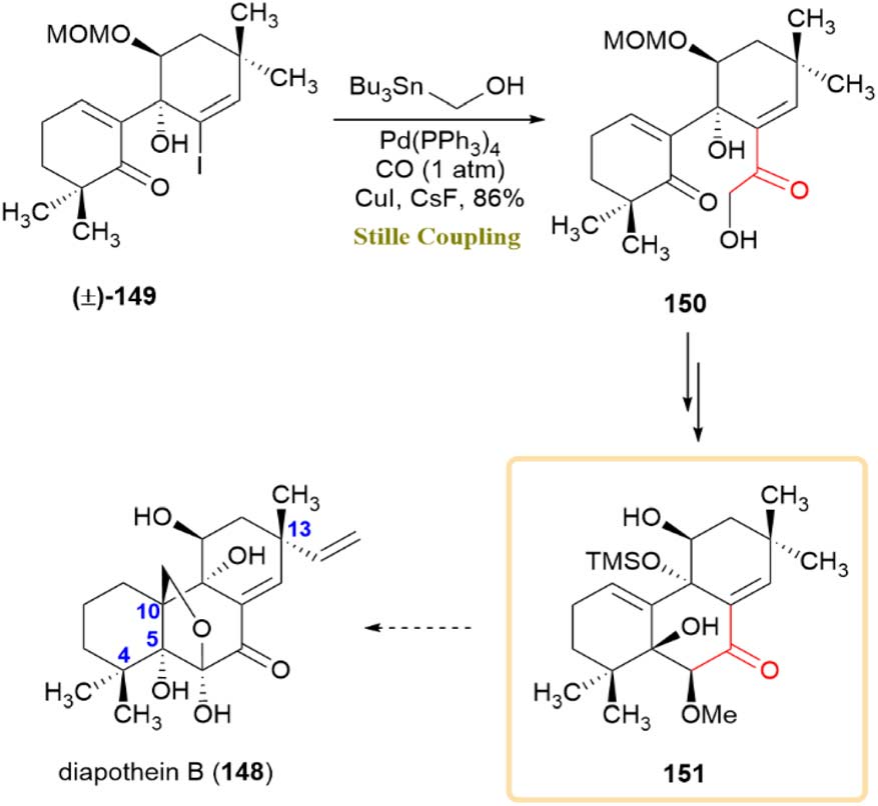
Carbonylative Stille coupling of vinyl iodide 149 furnished α-hydroxyketone 150, an advanced intermediate toward diaporthein B (148).

A synthetic strategy for the extensively oxidized carbon scaffold of diaporthein B (148) was developed through a 10-step sequence, employing convergent and diastereoselective fragment coupling techniques. This methodology successfully established the C4 and C13 quaternary centers, yet challenges emerged in forming the C10 quaternary center and achieving the desired stereochemistry at C5. A key transformation involved a carbonylative Stille coupling of vinyl iodide 149, yielding α-hydroxyketone 150 in a single step with an 86% yield (Scheme 38). The most advanced intermediate, compound 151, exhibited a highly oxidized tricyclic core resembling the carbon framework of 148. Although the total synthesis remained incomplete, the strategy provided a foundation for future synthetic efforts targeting 148 and related pimarane diterpenes.^77^

**Scheme 39:**
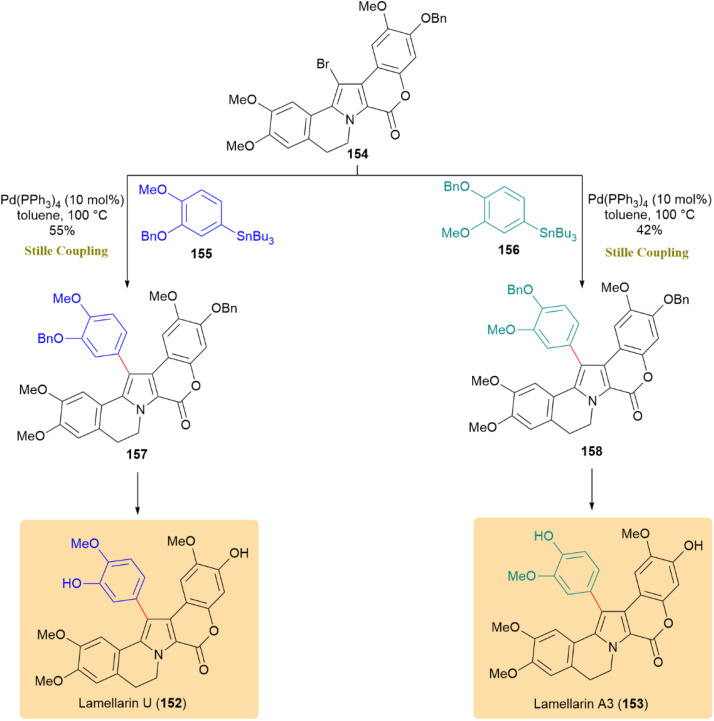
Compound 154 and aryl stannanes 155/156 coupled to give 157 and 158, completing lamellarins U (152) and A3 (153).

The total synthesis of lamellarins U (152) and A3 (153), marine-derived pyrrole alkaloids with potent cytotoxic and multidrug resistance reversal activities, was achieved *via* a late-stage Kosugi–Migita–Stille coupling. Compound 154 underwent Stille coupling with arylstannanes 155 or 156 using Pd(PPh_3_)_4_ (10 mol%) as the catalyst under standard conditions. The reaction process is illustrated in Scheme 39. This reaction produced compounds 157 and 158 in modest yields, accompanied by partial reductive debromination.^78^

**Scheme 40:**
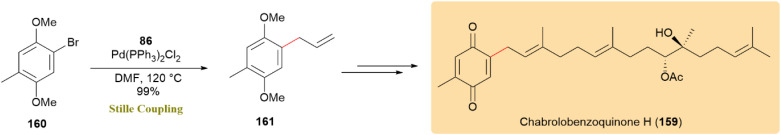
Bromide 160 and allyltributyltin 86 coupling led to formation chabrolobenzoquinone H (159).

Chabrolobenzoquinone H (159), a cytotoxic meroditerpene metabolite, was synthesized through a concise sequence in which a key Stille coupling joined bromide 160 with allylBu_3_Sn 86 using Pd(PPh_3_)_2_Cl_2_ and LiCl to afford the allyl derivative 161 in 99% yield. This transformation was strategically chosen because the Stille reaction tolerates the quinone and polyunsaturated functionalities of the meroditerpene framework, which are typically incompatible with other organometallic couplings. The use of LiCl enhanced transmetalation efficiency and minimized side reactions. Subsequent oxidative steps converted intermediate 161 into the target compound 159 (Scheme 40).^79^

**Scheme 41:**
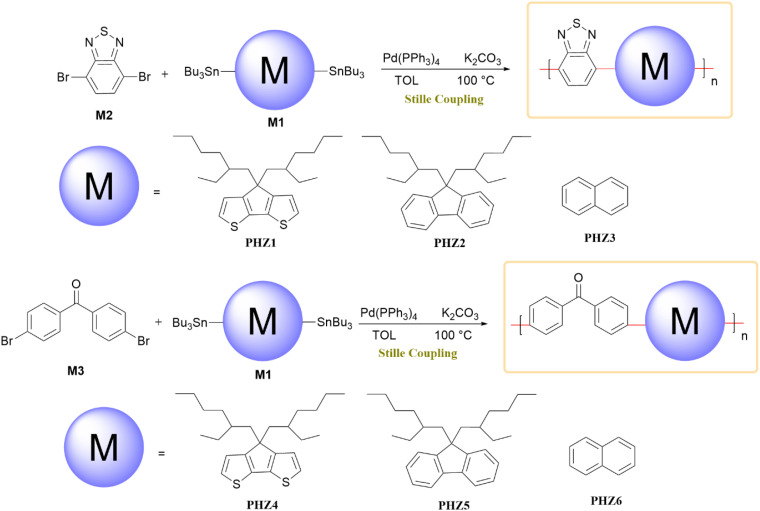
Monomer M1 and dibromides M2 and M3 coupled to yield oligomers PHZ1–PHZ6.

Six conjugated oligomers, designated PHZ1–PHZ6, were synthesized using the Stille coupling. They demonstrated excellent solubility in common solvents and exhibited distinct electrochromic color variations. The synthesis procedures, as outlined in Scheme 41, involved the reaction of monomer M1 with dibromides (M2 and M3) using K_2_CO_3_ and Pd(PPh_3_)_4_ in toluene under a nitrogen atmosphere for three days. After purification, the oligomers yielded PHZ1 (63%), PHZ2 (61%), PHZ3 (52%), PHZ4 (62%), PHZ5 (63%), and PHZ6 (58%). In this design, the Stille coupling played a crucial role in constructing well-defined donor–acceptor π-conjugated frameworks, enabling precise control over electronic communication between the benzothiadiazole or benzophenone acceptors and the thiophene-based donors. The reaction's mild conditions minimized side reactions and preserved functional integrity, offering advantages over other cross-coupling strategies which are less tolerant of heteroaromatic systems. This efficiency directly influenced the optical and electrochromic behaviors of the resulting oligomers.^80^

**Scheme 42:**
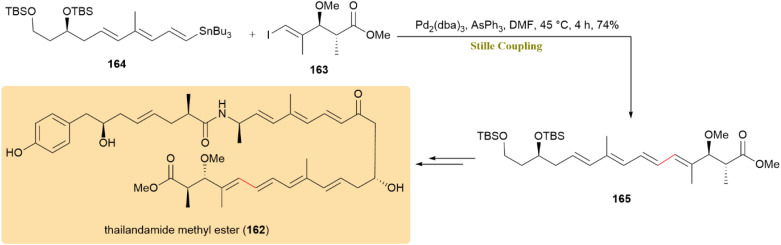
Stille coupling of vinyl iodide 163 and stannane 164 serves as a pivotal step in the synthesis of thailandamide A methyl ester (162).

A well-optimized Stille cross-coupling was reported as an essential step in the stereoselective synthesis of thailandamide A methyl ester (162), a complex polyene polyketide. Initial attempts were unsuccessful until the Stille coupling was employed. The vinyl iodide methyl ester 163 was efficiently coupled with organostannane 164 using Pd_2_(dba)_3_/AsPh_3_ in DMF, leading to the formation of compound 165, which was subsequently converted to product 162 (Scheme 42).^81^

**Scheme 43:**
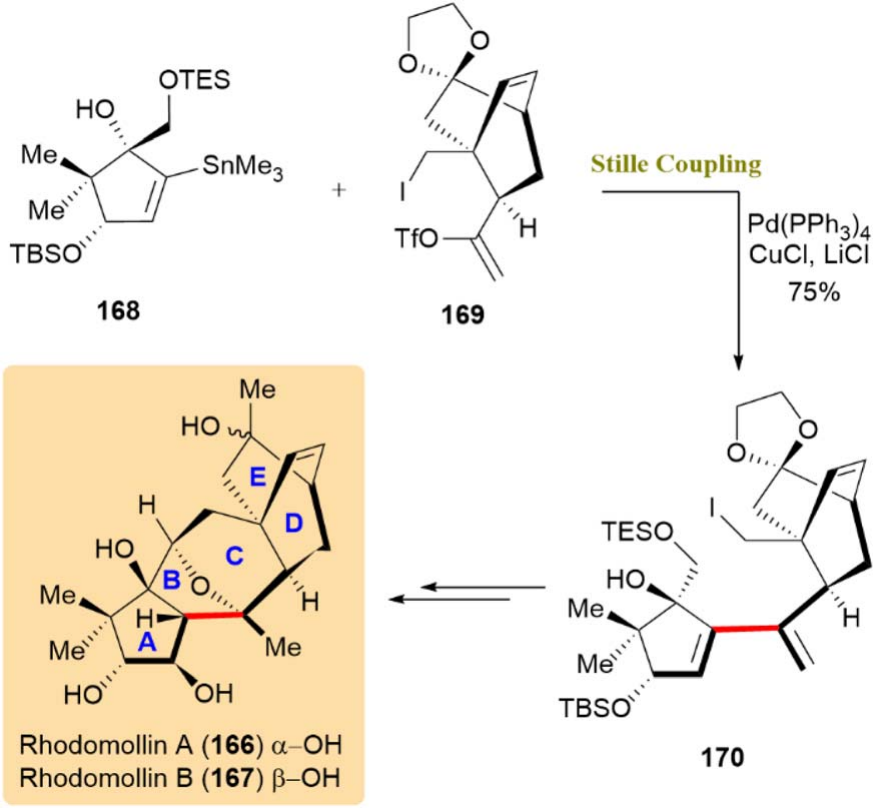
Fragments 168 and 169 coupled to form diene 170, key to rhodomollins A (166) and B (167).

The Stille coupling is essential a crucial role in the total synthesis of rhodomollins A (166) and B (167), as it is critical to constructing the tetracyclic carbon skeleton of these complex grayanoids. By coupling the A-ring fragment (compound 168), a stannylated intermediate, with the D/E-ring fragment (compound 169), a triflated intermediate, the reaction efficiently forms the conjugated diene intermediate (compound 170) in a 75% yield, setting the stage for subsequent transformations (Scheme 43).^82^

**Scheme 44:**
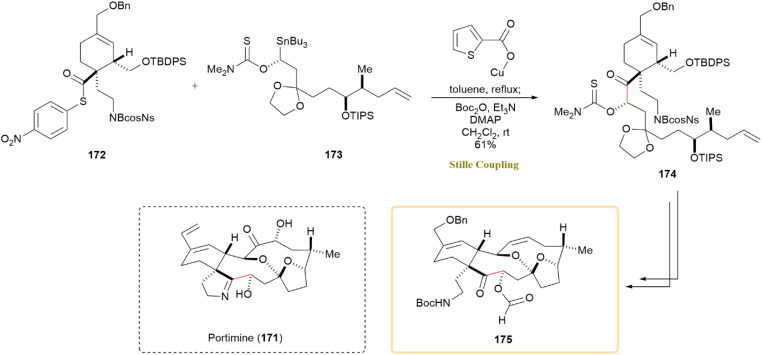
Thioester 172 and hiocarbamate 173 underwent CuTC-mediated Stille-like coupling to yield 174, precursor to portimine (171).

A synthetic approach toward the marine natural product portimine (171) was investigated, with a focus on a copper-mediated, stereoretentive C(sp^3^)–C(sp^2^) Stille-like coupling for assembling functionalized fragments. Unlike the traditional Pd-catalyzed Stille reaction, which primarily facilitates C(sp^2^)–C(sp^2^) couplings, the use of copper(i) thiophene-2-carboxylate (CuTC) as a catalyst enabled a stereospecific C(sp^3^)–C(sp^2^) coupling. This strategy follows the Falck/Liebeskind approach, which employs copper catalysis to achieve high stereochemical fidelity in couplings involving α-heteroatom-substituted alkylstannanes.^83,84^ The key reaction between thioester 172 and thiocarbamate 173 produced compound 174 with a 61% yield, retaining the chirality of α-alkoxyalkylstannane 173. The synthesis successfully produced alcohol 175, which retained the core skeleton of portimine. The reaction pathway is shown in Scheme 44. Further investigations are being pursued to finalize the total synthesis of portimine (171).^85^

In 2008, Fürstner developed a modified Stille–Migita cross-coupling utilizing [Pd(PPh_3_)_4_], CuTC, and [Ph_2_PO_2_][NBu_4_] as the catalytic system. This fluoride-free approach significantly improved reaction yields while preserving sensitive functional groups, like *O*-silyl and *C*-silyl groups, which are usually prone to degradation. The modification increased functional group tolerance and prevented undesired side reactions, making it particularly effective in complex natural product syntheses. Several challenging cross-coupling reactions were demonstrated, confirming the method's usefulness in palladium-catalyzed bond formation.^86^

**Scheme 45:**
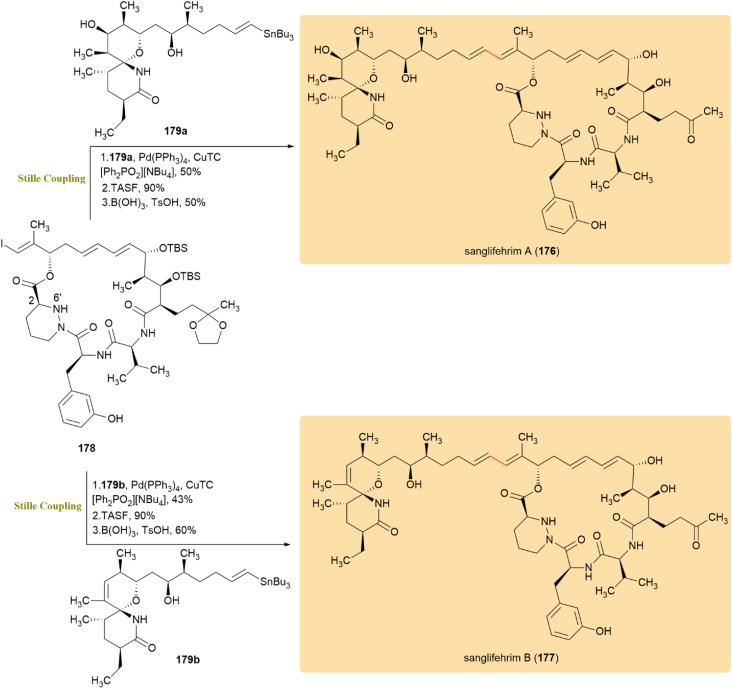
Vinyl iodide 178 and stannanes 179a/179b coupled to produce sanglifehrin A (176) and sanglifehrin B (177).

A convergent enantioselective strategy enabled access to sanglifehrin A (176) and the first complete synthesis of sanglifehrin B (177), two immunosuppressive macrocyclic natural products featuring a distinctive spirocyclic lactam. Vinyl iodide 178 was coupled with stannane 179a*via* the Stille–Migita protocol reported by Fürstner *et al.* to yield 176 in 50%, while 179b afforded 177 in 43% (Scheme 45). This cross-coupling represented a pivotal union step between the macrocyclic and spirolactam fragments, achieving chemoselective C–C bond formation under conditions mild enough to preserve the densely functionalized, stereochemically rich framework—an advantage over alternative coupling methods that are less compatible with nitrogen-rich macrocycles. The efficiency of this step facilitated late-stage diversification and enabled biological evaluation. Both 176 and 177 selectively bind cyclophilin A, with sanglifehrin B exhibiting greater antiproliferative potency in Jurkat T cells, highlighting their utility in studying protein–protein interactions and immunosuppres sion.^87^

**Scheme 46:**
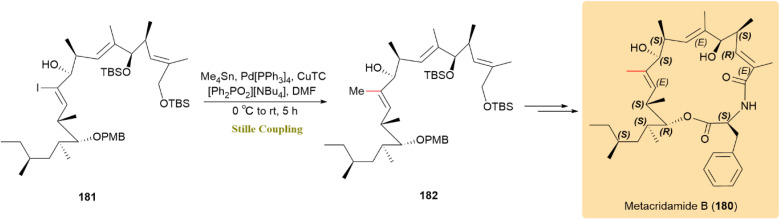
Iodide 181 and Me_4_Sn coupled to form 182, leading to metacridamide B (180).

The Stille reaction was used to synthesize metacridamide B (180), a cytotoxic 17-membered macrocyclic amide from *Metarhizium acridum*. This reaction forms carbon–carbon bonds between specific compounds. Its compatibility with various functional groups ensured successful bond formation, as demonstrated by an 84% yield in the coupling of iodide 181 and Me_4_Sn using the Fürstner method, resulting in the formation of compound 182 (Scheme 46). Metacridamide B (180) has shown activity against HepG2/C3A liver cancer cells, highlighting its potential in anticancer drug development.^88^

**Scheme 47:**
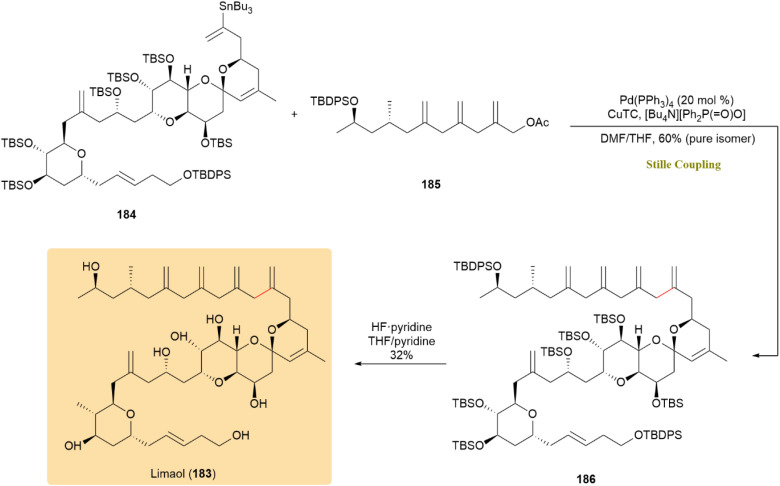
Fragments 184 and 185 coupled under Fürstner conditions to yield 186, completing limaol (183).

The comprehensive synthesis of limaol (183), a marine-derived C40-polyketide, was described, with a focus on its distinctive feature of four skipped methylene groups in the hydrophobic tail. As shown in Scheme 47, the tail region, which contains these “exo”-methylene groups, was successfully incorporated through palladium-catalyzed fragment coupling of compounds 184 and 185 using Fürstner conditions. This approach yielded compound 186 with a 60% pure isomer yield, resulting in the successful synthesis of 183.^89^

**Scheme 48:**
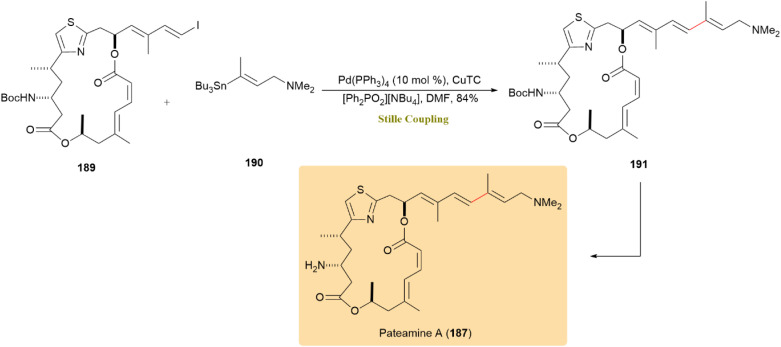
Alkenyl iodide 189 and stannane 190 coupled to form intermediate 191, converted to pateamine A (187).

Efficient management of protecting groups a modern Stille coupling protocol enabled the synthesis of pateamine A (187) and its analogue DMDA-Pat A (188). These compounds exhibit significant anticancer activity, with DMDA-Pat A also showing potential for reducing tumor growth and preventing of cancer-related muscle loss. The reaction of alkenyl iodide 189 with stannane 190, using the Fürstner method, yielded compound 39 with 84% efficiency, which was then converted to Pateamine A (187) (Scheme 48).

**Scheme 49:**
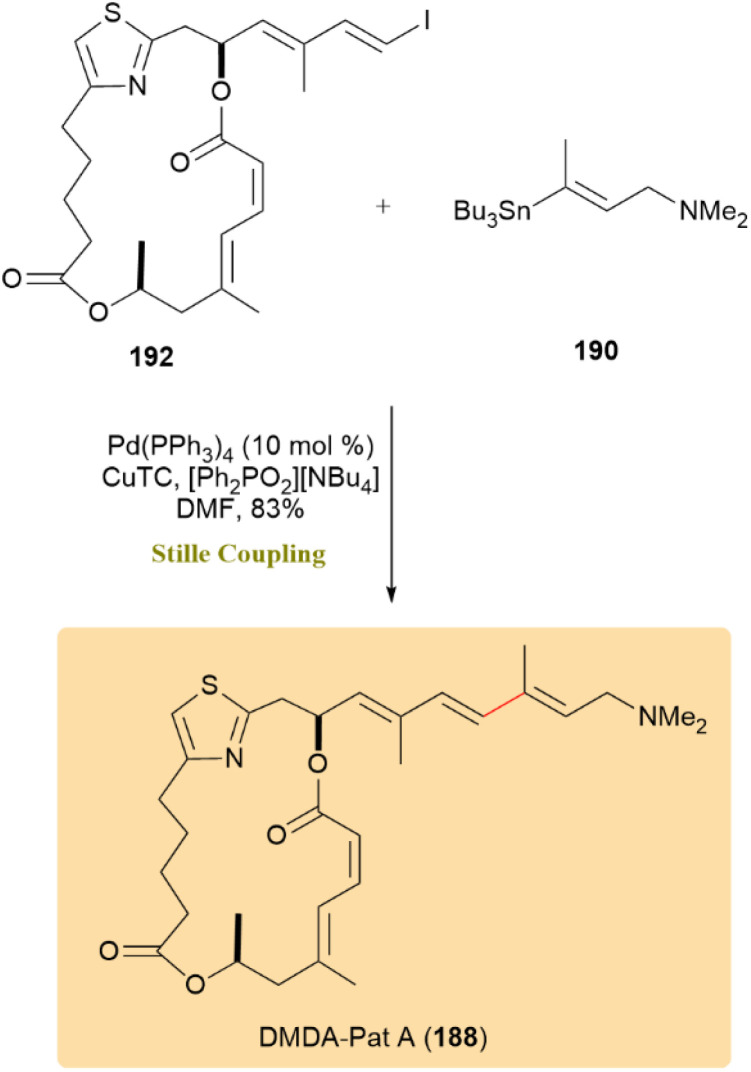
Compound 192 and stannane 190 coupled to give 193, precursor to DMDA-Pat A (188).

Similarly, coupling compound 192 with stannane 190 produced DMDA-Pat A (188) with 83% yield (Scheme 49).^90^

**Scheme 50:**
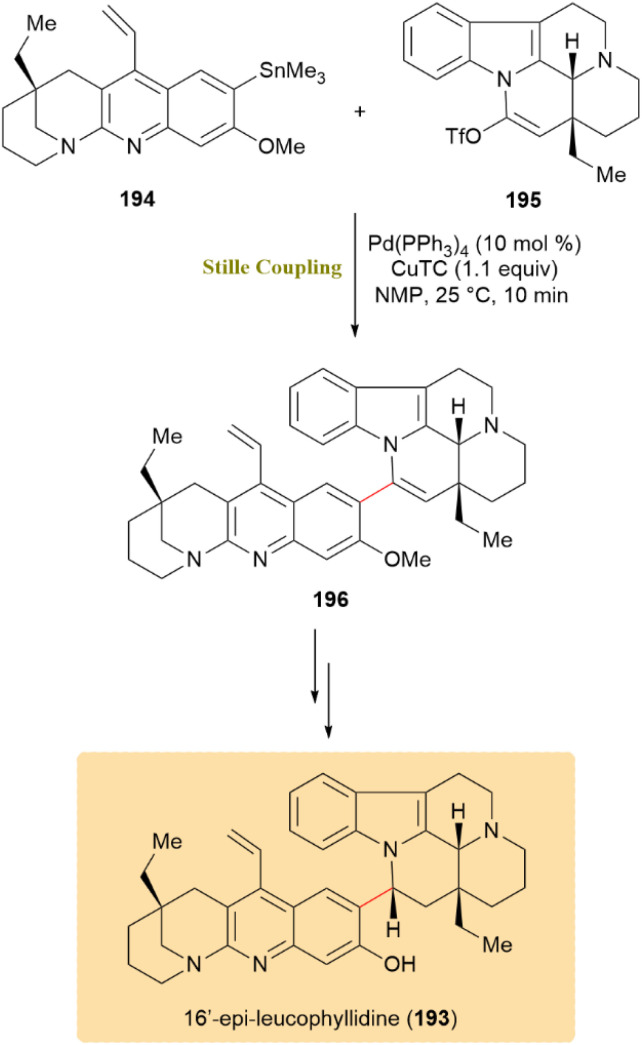
Stille coupling of trimethylstannane 194 and triflate 195 serves a pivotal step in the synthesis of 16′-*epi*-leucophyllidine (193).

As shown in Scheme 50, the total synthesis of 16′-*epi*-leucophyllidine (193), a synthetic analogue of the cytotoxic alkaloid leucophyllidine, was facilitated by the efficient cross-coupling of trimethylstannane 194 with triflate 195, employing Pd(PPh_3_)_4_ and copper(i) thiophene-2-carboxylate (CuTC), inspired by Fürstner modification, resulting in dimer 196 with a 69% yield after 10 minutes. This transformation served as the key bond-forming step, efficiently uniting two highly functionalized indole fragments under exceptionally mild conditions. The use of CuTC accelerated the transmetalation step, allowing rapid coupling while preserving the sensitive indole and amide functionalities—a hallmark advantage of the Stille–Fürstner protocol in complex alkaloid synthesis. The resulting 16′-*epi*-leucophyllidine (193) retained the biological activities of leucophyllidine, including cytotoxic and nitric-xide-synthase-inhibitory properties.^91^

**Scheme 51:**
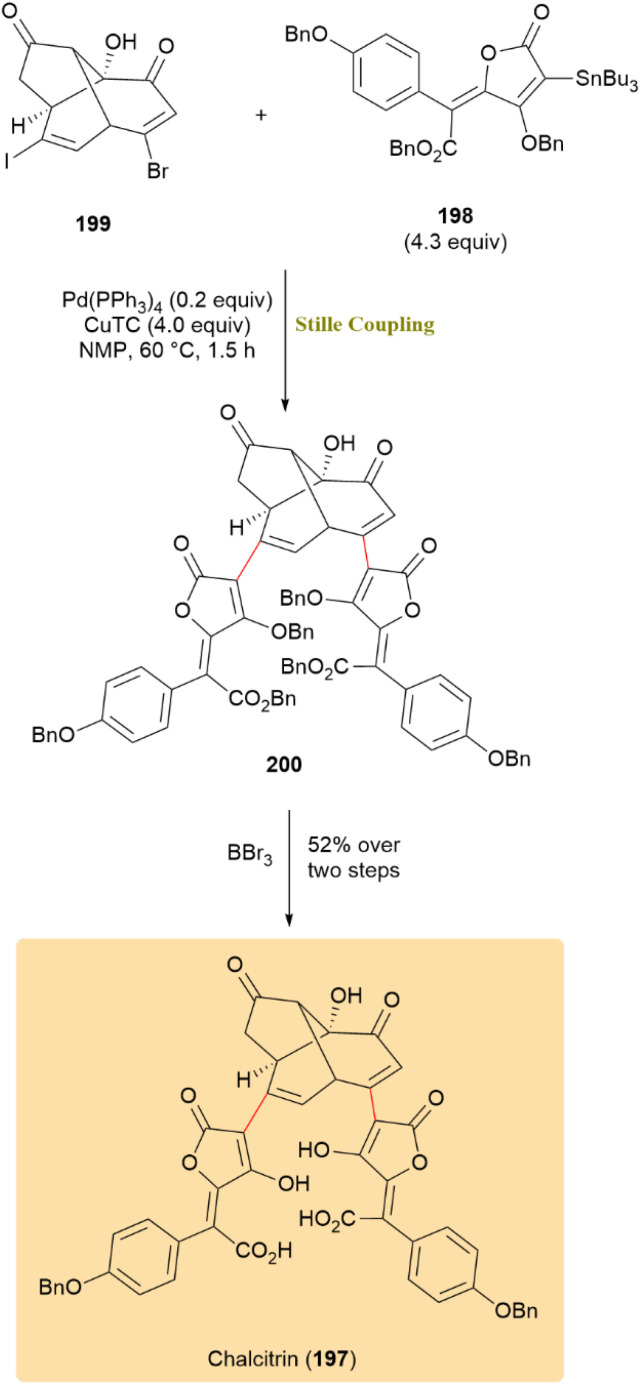
Double Stille coupling of stannyl 198 with vinyl halide 199 formed 200, leading to chalcitrin (197).

With similar condition, the first accomplished total synthesis of chalcitrin (197), a rare fungal pigment and pulvinic acid dimer with potential metal-ion binding properties, was achieved through a double Stille coupling at the late stage. Stannyl 198 was successfully coupled with double vinyl halide 198 at both reactive sites using catalytic Pd(PPh_3_)_4_ and CuTC, forming compound 200. Subsequent deprotection of all six benzyl groups with excess BBr_3_ produced 197 in 52% yield for the last two steps (Scheme 51).^92^

**Scheme 52:**
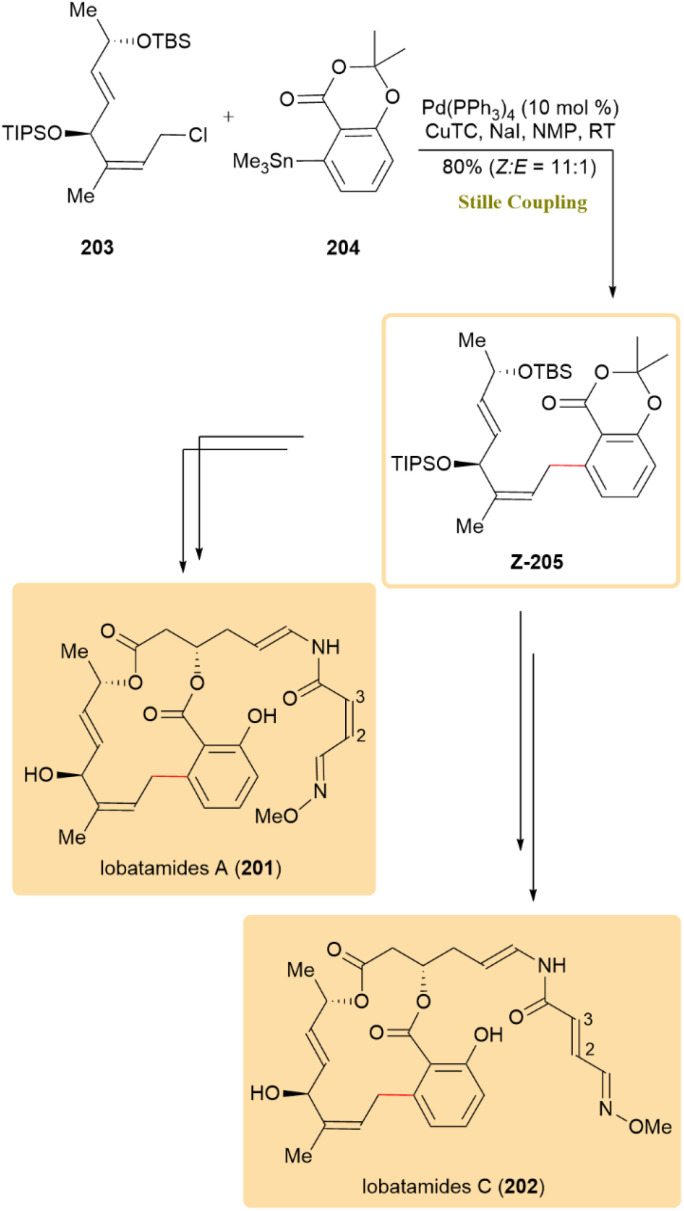
Allylic chloride 203 and aryl stannane 204 coupled to give *Z*-205, precursor to lobatamides A (201) and C (202).

The complete synthetic route for lobatamides A (201) and C (202) was reported, with particular focus on the formation of their macro-benzolactone and enamide side chains, both known for V-ATPase inhibition and antitumor activity. The two compounds differ only in the geometry of the C2–C3 double bond in the enamide: 201 has a (*Z*,*E*)-configuration, while lobatamide 202 has an (*E*,*E*)-configuration. A key allylic aryl structure was constructed using a Migita–Kosugi–Stille coupling. In this step, allylic chloride 203 was coupled with aryl stannane 204 under conditions similar to those reported by Fürstner, employing 10 mol% Pd(PPh_3_)_4_, CuTC, and NaI in NMP. This reaction produced the allylic aryl intermediate *Z*-205 in 80% yield with a *Z* : *E* ratio of 11 : 1 (Scheme 52).^93^

## 3. The intramolecular Stille coupling

**Scheme 53:**
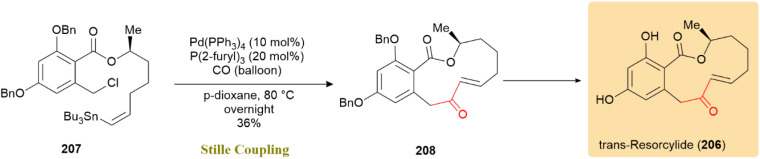
Vinylstannane 207 underwent carbonylative Stille coupling to form *trans*-enone 208, completing *trans*-resorcylide (206).

The total synthesis of *trans*-resorcylide (206), a bioactive member of the resorcylic macrolide family with plant growth inhibitory, antifungal, and cytotoxic properties, was achieved *via* a palladium-catalyzed Stille carbonylation. This key step constructed the 12-membered macrocycle by converting *cis*-vinylstannane intermediate 207 into *trans*-enone 208 using 10 mol% Pd(PPh_3_)_4_ and 20 mol% P(2-furyl)_3_, affording the product in 36% yield (Scheme 53). This reaction not only represents the first example of a macrocyclic Stille carbonylation applied to a benzyl chloride substrate but also provided an efficient alternative to traditional macrolactonization or ring-closing metathesis strategies, which often fail under such sterically constrained and functionalized conditions. The transformation proceeds through an intramolecular carbonylative coupling that constructs the macrocyclic enone with complete chemoselectivity, underscoring the power of the Stille carbonylation in complex macrocycle formation.^94^

**Scheme 54:**
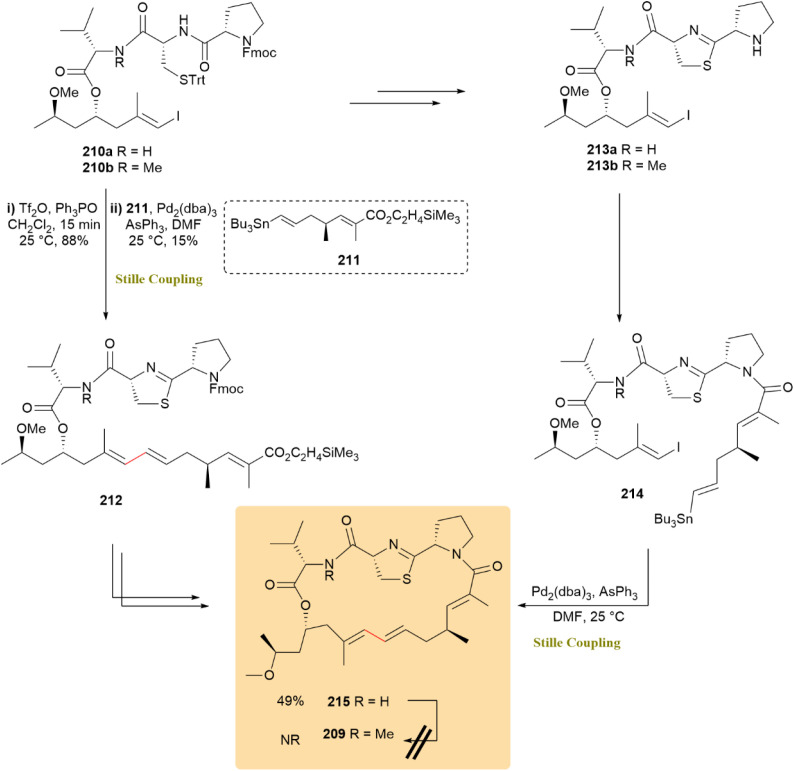
Stille coupooling of iodide 210a, yielded compound 215, an advanced intermediate toward alotamide A (209).

The synthesis of the neurotoxin alotamide A (209) was investigated by employing an intramolecular Stille reaction (C sp^2^–C sp^2^) along with a macrolactam-forming step. An advanced intermediate was prepared, leading to the development of an efficient stereoselective synthesis protocol for the polyketide fragment, although complete synthesis was not achieved. Iodide 210a was coupled with stannane 211, yielding product 212 at 15% using Pd_2_(dba)_3_ and AsPh_3_. Another route involved treating iodide 210a to produce 213a and then intermediate 214, followed by intramolecular Stille cross-coupling, which isolated compound 215 with a 49% yield (Scheme 54).^95^

**Scheme 55:**
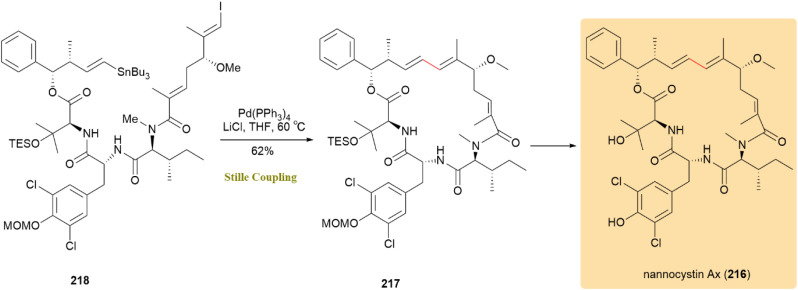
Afforing nannocystin Ax (216) *via* intramolecular Stille coupling.

A complete synthesis of nannocystin Ax (216) was reported, highlighting the construction of a distinctive 21-membered macrocyclic core with over seven stereocenters, incorporating tripeptide and polyketide elements. Biological studies exhibited potent cytotoxic activity against a range of tumor cell lines and a strong antifungal effect by nannocystin A. Compound 217 was synthesized from compound 218 in a 62% yield *via* intramolecular Stille cross-coupling in dilute THF with Pd(PPh_3_)_4_ and LiCl at 60 °C, enabling the completion of product 216 synthesis (Scheme 55).^96^

**Scheme 56:**
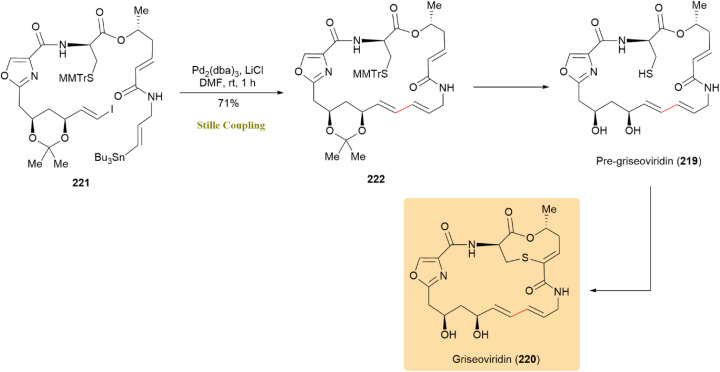
Compound 221 underwent intramolecular Stille coupling to form macrocycle 222, precursor to griseoviridin (220).

The first total synthesis of pre-griseoviridin (219), which was later converted to griseoviridin (220), a broad-spectrum antibacterial group A streptogramin from Streptomyces, was achieved through an intramolecular Stille reaction. Compound 221 participated in this reaction with Pd_2_(dba)_3_ and LiCl, yielding macrocycle 222 with a 64% yield. Initial deprotection efforts were hindered by the stability of the trityl group; however, replacing it with a 4-monomethoxytrityl (MMTr) group and adding LiCl improved the yield to 71% (Scheme 56).^97^

**Scheme 57:**
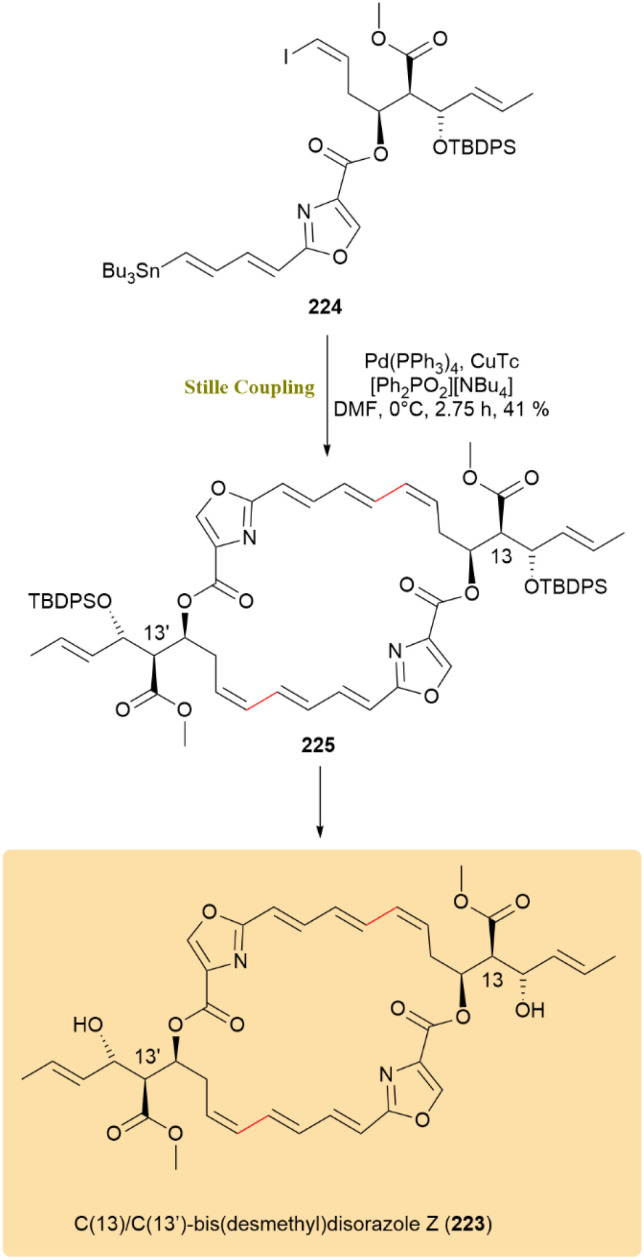
Double inter-/intramolecular Stille coupling of precursor 224 to afford disorazole Z analogue 223.

The synthesis of the bioactive disorazole Z analogue, C(13)/C(13′)-bis(desmethyl)disorazole Z (223), known for its microtubule-disrupting and anticancer effects, was completed *via* total synthesis. A key transformation in the synthetic route was the application of the Fürstner method in an intramolecular Stille coupling, highlighting its utility in complex macrocycle formation. The use of double inter- and intramolecular Stille cross-coupling in the cyclodimerization of a bifunctional vinyl stannane/iodide precursor represented a significant advancement in macrodiolide synthesis. Under Fürstner's condition at 0 °C, stannyl vinyl iodide 224 underwent a double inter-/intramolecular Stille reaction to obtain macrodiolide 225 in 41% yield, with preservation of the triene configuration and no observable isomerization (Scheme 57).^98^

## 4. Conclusions

Over the past decade, the Stille coupling has continued to evolve as a crucial and versatile method in the synthesis of natural products. Its exceptional tolerance for diverse functional groups, compatibility with a wide range of substrates, and mild operating conditions have allowed for the efficient creation of intricate molecular structures with high stereo- and regioselectivity. Recent advancements such as bimetallic and heterogeneous catalysis, copper co-catalyzed systems, and new ligand designs have significantly broadened the reaction's capabilities, enhancing yields, selectivity, and suitability for more challenging substrates. Despite these developments, the field is increasingly focused on addressing environmental and safety concerns. The dependence on organotin reagents and difficulties in catalyst recovery and metal leaching remain significant limitations, encouraging the development of greener, tin-free, and recyclable catalytic systems. Although organotin toxicity presents challenges, organostannanes still offer valuable reliability in complex coupling processes. Continued progress in enantioselective protocols and environmentally considerate methods underscores the ongoing evolution of the Stille reaction. Demonstrated through numerous successful syntheses of marine alkaloids, diterpenoids, macrolides and complex polyenes, the Stille coupling remains a fundamental aspect of modern synthetic strategies, ready to meet future challenges in the construction of complex molecules and sustainable chemistry.

## Conflicts of interest

There are no conflicts to declare.

## Data Availability

No new experimental data were generated in this study. This review is based entirely on previously published literature, and all cited sources are properly referenced in the manuscript.
